# Joint meeting of the British and European Associations for Cancer Research and the Royal Society of Medicine (Section of Oncology). November 3-5, 1986, London. Abstracts.

**DOI:** 10.1038/bjc.1987.65

**Published:** 1987-03

**Authors:** 


Br. J. Cancer (1987), 55, 331–349 © The Macmillan Press, Ltd., 1987

### Joint Meeting of the British and European Associations for Cancer Research and the Royal Society of Medicine (Section of Oncology)*

(Incorporating Symposia on 'Lung Cancer' and 'Lymphoma', the 6th Alexander Haddow Memorial Lecture and the 7th Gordon Hamilton-Fairley Memorial Lecture) November 3–5, 1986.

*Held at the Royal Society of Medicine, 1 Wimpole Street, London W1.*

### Abstracts of Invited Papers†

### Symposium on Lung Cancer

### The endocrinology of lung cancer

K. Havemann & G. Bepler

*Department of Medicine, Division of Hematology/Oncology, University of Marburg, 3550 Marburg, FRG.*

Small cell lung cancer (SCLC) is thought to be derived from neuroendocrine cells of the bronchopulmonary tract. This assumption is based on morphological features, i.e. dense core vesicles, and biochemical characteristics, i.e. the production of neuroendocrine peptides and the expression of enzymes of the diffuse neuroendocrine system. As a result, SCLC has been classified as a malignant neuroendocrine tumour of the lung. We have studied the production of bombesin, neurotensin, calcitonin, calcitonin gene-related peptide (CGRP), somatostatin and tachykinins in 12 SCLC and 8 non-SCLC cell lines. Bombesin, neurotensin, calcitonin, CGRP, somatostatin and tachykinins were produced by some but not all SCLC cell lines. Non-SCLC lines had undetectable levels. Neuroendocrine enzymatical markers such as NSE or CK-BB were present in all lung cancer cell lines but significantly higher in SCLC. Specific membrane binding sites for bombesin and calcitonin were found in SCLC but not in non-SCLC cell lines. The production of peptides and expression of membrane binding sites suggest an autocrine effect on tumour growth. The peptides were thus screened for their growth in liquid culture and semi-solid agarose culture using serum-free techniques. Bombesin was found to stimulate the growth of 8 independent SCLC cell lines in a serum-free cloning system only. This effect was absent in the presence of a bombesin receptor antagonist. In addition, we evaluate the usefulness of calcitonin, neurotensin, NSE and CK-BB as tumour markers in sera of SCLC patients who entered a prospective multicentre trial. Our results indicate that the levels of the peptide and enzyme markers correlate with tumour spread, prognosis, and early response to therapy.

### The cell surface in lung cancer

J. Bergh

*Department of Oncology, University of Uppsala, S-751 85 Uppsala, Sweden.*

Human small cell carcinoma of the lung (SCCL) is characterized by a spectrum of neuroendocrine markers. Non-SCCL on the other hand is deprived of these characteristics, or express them in low quantities. The cell surface is of major importance for inter- and intracellular communications via specific receptors and transport mechanisms. The surface membrane is composed of a vast number of glycoproteins (GP) and glycolipids (GL), but their specific functions are still mainly unknown.

The descriptive analyses of the surface GP using the galactose-oxidase tritiated sodium borohydride technique and $^{125}I$ labelling techniques have demonstrated that SCCL expresses a different pattern of surface GP as compared to non-SCCL. The principal patterns of these GP also seem to remain unaltered during prolonged *in vitro* cultivation and upon growth in defined medium. The surface GL of SCCLs contain a specific monosialoganglioside, fucosyl-$GM_1$, and 2-hydroxy-fatty acids as major gangliosides in high frequency of examined biopsies. In contradistinction to the surface GP, these 'specific' GL seem to be altered during prolonged *in vitro* cultivation of SCCL cell lines.

### Lung tumour markers

J.G. Reeve

*MRC Clinical Oncology and Radiotherapeutics Unit, MRC Centre, Hills Road, Cambridge CB2 2QH, UK.*

Much research has been devoted to the biochemistry of lung cancer cells with a view to identifying markers for clinical application and for tracing cell lineage relationships between the major forms of lung cancer. The endocrine status of small cell lung cancer (SCLC) has led to the evaluation of peptide and non-peptide hormones, enzymes and structural proteins as diagnostic markers for the SCLC phenotype. However, it is now generally accepted that many of these substances are associated with all the histological types of bronchogenic carcinoma. Whilst the presence or absence of a given biochemical marker may be of little diagnostic value, certain biomarkers may be useful for monitoring therapy particularly in patients with SCLC where a relationship between tumour burden and levels of NSE, calcitonin and ACTH has been demonstrated. However, there exists no single marker for which elevated levels can be measured in all patients with lung cancer and in many recurrences, markers rise indistinctly or too late to be clinically useful, or not at all. The search for reliable lung tumour markers has prompted many groups to develop monoclonal antibodies (MoAbs) to membrane determinants. A number of MoAbs now exist which are histologically type-specific and others recognise antigens common to SCLC and non-SCLC, perhaps defining a phenotypic link between these two histologies. Many MoAbs fail to fulfil the clinical objectives

*BACR enquiries to: BACR Secretariat, c/o Institute of Biology, 20 Queensberry Place, London SW7 2DZ.

†Reprints of these abstracts are not available – Ed.

for which they were developed. However, several are likely to be of diagnostic value, some detect shed tumour-antigens and may be of value in monitoring therapy and others are selectively cytotoxic to tumour cells bearing the defined antigen or are antiproliferative. In some cases clinical evaluation of such MoAbs is in progress.

### Genetic predisposition to human lung cancer

J. Heighway

*Paterson Institute for Cancer Research, Christie Hospital, Manchester M20 9BX, UK.*

A restriction fragment length polymorphism (RFLP) has been shown to be associated with the human c-Ha-*ras* gene. The RFLP has been shown to result from the variation in size of a region of repetitive DNA, approximately 1kb 3′ of the polyadenylation signal of the gene. Four common alleles have been identified (a1 to a4) with occasional rare variants and Krontiris *et al* (1985) *Nature* **313**, 369 suggested that rare alleles predisposed an individual to cancer.

The c-Ha-*ras* allele frequency has been studied in a group of lung cancer patients and unaffected controls. The patients were divided into two groups (a) individuals with small cell carcinoma (SCCL) (b) those with non-small cell carcinoma (N-SCCL). No significant variation in the frequency of rare alleles was found between the groups of cancer patients and unaffected controls. However a significant difference in the frequency of one allele (a4) was found between individuals with N-SCCL and unaffected controls ($P<0.05$) and between those with N-SCCL and SCCL ($P<0.004$) This suggests a degree of genetic predisposition to N-SCCL.

### 6th Alexander Haddow Memorial Lecture

### The molecular biology of lung cancer

J. Minna

*NCI-Navy Medical Oncology Branch, US National Cancer Institute, Bethesda, USA.*

Molecular genetic studies on a large panel of human small cell lung cancer (SCLC) lines have revealed a consistent set of abnormalities that could potentially explain part or all of the malignant phenotype of these cells. *First,* restriction fragment polymorphism analysis (by Dr S. Naylor, Univ. Texas) has demonstrated that the cytogenetic deletion of chromosome region 3p (14–23) represents a true DNA deletion. This suggests SCLC may have similar genetic mechanisms operating, as those in retinoblastoma and Wilm's tumours. *Second,* frequently we have found amplification or deregulated expression of the *myc* family of oncogenes (including c-, N-, and L-*myc*) as well as expression of the p53 proto-oncogene. The structure and expression of L-*myc* are particularly interesting as alternative processing of L-*myc* mRNA is seen in both small cell and non-small cell lung cancer groups. This alternative processing generates messages with different combinations of the 2nd and 3rd exon *myc* family equivalents, a feature not yet described for c-*myc* or N-*myc*. In addition, expression of cellular proto-oncogenes of the *ras* and *raf* (studies by Dr U. Rapp, NCI) families in these same cells set the stage for oncogene cooperation. *Finally,* it appears that both SCLC and non-SCLC lines can replicate in serum- and hormone-free medium for prolonged periods of time indicating their ability either to produce autocrine growth factors (such as gastrin releasing peptide) or subvert intracellular transducing signals to function in an autocrine fashion. (Abstract only.)

### The current status of anti-smoking measures in the UK

D. Simpson

*Action and Smoking and Health, 5–11 Mortimer Street, London W1N 7RH, UK.*

Despite having led the world in the epidemiology of smoking and still having the highest death rate from lung cancer in the world, the UK has a far from satisfactory record in the implementation of effective anti-smoking measures. It was one of the first countries to implement such measures, but still has no overall government policy on smoking and the health department policy is not comprehensive and falls far short of standards set by WHO, UICC, the Royal College of Physicians and other agencies. Nevertheless, the present decline in consumption is among the fastest in the world. An ideal smoking control policy includes action in the areas of health education, public information, tobacco promotion, smoking in public places, taxation and the reduction of emission levels of tar, carbon monoxide and nicotine. Outstanding in recent years has been the UK's performance on the increase of the real price of cigarettes by means of regular tax rises. Performance has been disappointing, however, in restricting advertising and other forms of promotion in public places, both energetically resisted by the tobacco industry. Health education has been low in status, priority and expenditure but public information work through the news media has been comparatively successful.

### Advances in the clinical therapy of lung cancer

R.L. Souhami

*Department of Radiotherapy and Oncology, University College Hospital, Gower Street, London WC1E 6AU, UK.*

Combination chemotherapy has improved median survival in small cell lung cancer (SCLC) but the proportion of patients surviving beyond 2 years is less than 10%. Attempts to improve results by alternating drug regimens have not been successful, but active new agents such as carboplatin, ifosfamide and VM26 have yet to be incorporated into multiagent regimens. The optimum duration of combination chemotherapy has not been determined. Recent studies have identified patients who have a greater than 25% chance of survival beyond 2 years, in whom intensification of chemotherapy may increase cure rate. Patients with multiple adverse prognostic factors may have their survival shortened by the use of combination chemotherapy. Such patients can be identified and alternative treatment strategies designed to minimise toxicity, can be developed. Mediastinal irradiation may benefit a small number of patients who have a complete response to chemotherapy. Prophylactic cranial irradiation does not appear to prolong survival but may be justified by the unpleasant consequences of brain metastasis.

In non small cell lung cancer (NSCLC) it is not known whether mediastinal irradiation improves the prognosis of patients with resectable tumours but who have mediastinal node involvement. In advanced NSCLC there is a lack of effective drugs but response rates of 20–30% can be achieved with several combinations. There is no overall survival benefit from chemotherapy, but patients can be identified who have a greater chance of response and it remains to be shown if they benefit from treatment.

### Symposium on Lymphoma

### Retroviruses and lymphoma

R.A. Weiss

*Institute of Cancer Research: Royal Cancer Hospital, Chester Beatty Laboratories, Fulham Road, London SW3 6JB, UK.*

Retroviruses are well known aetiological agents of lymphoma in fish, birds and mammals. Since 1980, retroviruses have been identified in association with lymphoma in man. Human T-cell leukaemia virus type I (HTLV-I) is the principal cause of adult T-cell leukaemia-lymphoma (ATLL), a malignancy of mature T4 lymphocytes prevalent in Japan and the Caribbean. Lymphomas are also the second commonest tumour in AIDS, a syndrome caused by another kind of retrovirus, the human immunodeficiency virus type (HIV-1). In ATLL, the retrovirus directly induces T-cell transformation leading to eventual malignancy, whereas in AIDS, the lymphomas are generally EBV-positive B-cell neoplasms probably arising as a result of immunosuppression induced by HIV-1. Current ideas on the molecular mechanisms of cell transformation and immunosuppression by human retroviruses will be reviewed.

### Epstein–Barr virus and lymphoma

A.B. Rickinson

*Department of Cancer Studies, University of Birmingham Medical School, Birmingham, B15 2TJ, UK.*

Epstein–Barr virus (EBV)-associated lymphomas can now be divided into two categories. The EBV-positive lymphoproliferations seen most commonly in immunosuppressed transplant patients are oligoclonal, carry no specific chromosomal translocations and appear, from clinical evidence, to remain sensitive to virus-specific T cell control; experimental EBV infection of cotton-top marmosets induces a very similar disease. By contrast EBV-positive Burkitt's lymphomas (BL) seen most commonly in equatorial Africa (but occurring worldwide) are monoclonal, carry specific translocations leading to constitutional activation of the c-*myc* gene, and are usually not recognised by virus-specific T cell surveillance.

We believe these differences relate in part to the separate identities of the target cells involved, and to their different interactions with the virus. In particular surface marker analysis of BL biopsies has revealed a homogeneous BL cell phenotype positive for pan B markers, for CD10 (cALLA) and for the tumour-associated glycolipid antigen BLA, but negative for the various B cell activation antigens seen on all normal EBV-transformed lymphoblastoid cell lines (LCLs). It is significant that tumour cell lines which retain the BL cell phenotype *in vitro* show a unique pattern of expression of EBV latent genes which is more restricted than that shown in LCLs. This new form of infection may be a unique feature of the malignant cells or may reflect the usual interaction of the virus with the particular B cell subset from which BL arises. We have now identified and isolated a normal tonsillar B cell subpopulation with the same cell surface phenotype as the tumours and are studying its interaction with EBV.

### Phenotypes of anaplastic large cell lymphomas

G. Delsol

*Department of Pathology, CHU Purpan, Toulouse 31059, France.*

Among large cell lymphomas, a distinctive group of 65 cases with very large anaplastic cells, often unfiltrating the lymph node sinuses, was identified. According to the reactivity of these tumours with monoclonal antibodies (MoAbs) detecting epithelial membrane antigen (EMA) and the recently described Kil antigen, four subtypes could be distinguished. In the majority of cases ($n=48$), neoplastic cells were found to coexpress EMA and Kil antigen. All the cases studied on frozen sections also proved to be positive for three anti-Tac antibodies (type 1: EMA+, Ki+, Tac+). In the second type ($n=7$) neoplastic cells expressed Kil antigen but were unreactive with the anti-EMA and anti-Tac MoAbs (type 2: EMA−, Kil+, Tac−). There were also tumours with similar morphology expressing only EMA (type 3: EMA+, Kil−, Tac?) ($n=4$) or with an EMA−, Kil−, Tac− phenotype (type 4: 6 cases). Whether these various phenotypes have prognostic significance cannot yet be determined. MoAbs identifying T, B, or macrophage-associated antigens showed that these cases were heterogeneous in terms of cell lineage. However, for the tumours coexpressing EMA, Kil and Tac antigens the commonest origin was T cell (15 cases), no definite cell lineage could be attributed to 11 cases; only 3 cases showed B cell markers and 3 other cases displayed a mixed T/B phenotype. By contrast, tumours of type 2, 3 or 4 were mainly developed from B cells ($n=10$) or showed a mixed B/T ($n=2$) or nul ($n=2$) phenotype. Although morphological characteristics of the majority of cases were consistent with the diagnosis of malignant histiocytosis, double immunoenzymatic staining showed clearly that EMA+, Kil+ neoplastic cells were unreactive with anti-macrophage antibodies. These results confirm that true malignant histiocytoses are very rare.

### Growth factors and their receptors

T.M. Dexter

*Paterson Institute for Cancer Research, Christie Hospital and Holt Radium Institute, Manchester M20 9BX, UK.*

### 7th Gordon Hamilton-Fairley Memorial Lecture

### Oncogene activation by chromosomal translocation in carcinogenesis

G. Klein

*Department of Tumour Biology, Karolinska Institutet, Box 60400, S-104 01 Stockhom, Sweden.*

Current evidence concerning the mechanism of oncogene activation by chromosomal translocations and its role in various forms of tumorigenesis will be reviewed. Specific attention will be devoted to the role of the c-*myc*/Ig juxtaposition in the genesis of Burkitt lymphoma, mouse plasmacytoma and rat immunocytoma. The following aspects will be discussed: (i) Does the *cis*-relationship between the c-*myc* oncogene and one of the 3 Ig-loci play a causative role in the genesis of these tumours? (ii) How does the juxtaposition activate the *myc*-gene? (iii) What is the functional role of the translocation in the tumorigenic process?

In BL, the translocation has been found in 100% of the properly investigated cases so far, with no difference between endemic or nonendemic, EBV carrying or EBV negative cases. In MPC, only ~90% of the MPCs carry the translocations. Cytogenetic and molecular examination of the exceptional tumours has revealed, however, that they carry cryptic Ig-*myc* juxtapositions. The regularity of the association between the translocation events and the tumours where they occur, together with the analogy between the 3 systems can only be interpreted by postulating that the activation of c-*myc* by the translocation represents an essential step in the genesis of BL, MPC and RIC. A hypothesis will be presented to explain the way in which the constitutive *myc* activation contributes to the escape of the tumour precursor cells from immune and non immune controls. (Abstract only.)

### Gene probing as a criterion of monoclonality

K. Thielemans

*Division of Hematology and Immunology, Medical School of the Free University of Brussels (VUB), 1090 Brussels, Belgium.*

A molecular genetics approach to the diagnosis of lymphoid proliferative disorders, providing a sensitive marker for clonality as well as for lineage assignment (B *vs.* T lymphocytes) in neoplasms lacking definitive phenotypic markers, has been developed recently. This approach relies on detecting uniform rearrangements of the genes coding the antigen recognition molecules of B cells (immunoglobulin) and T cells (T cell receptor) within clonal proliferations. The analysis of the Ig and TCR genes by Southern blot hybridisation has great potential for complementing conventional marker analysis, cytogenetics and histopathology in the definition, classification and diagnosis of lymphoproliferative disorders. It becomes a useful tool with which to follow the patient's therapeutic response and monitor the patient's clinical course for early signs of relapse. Molecular genetic analysis, in association with cytogenetics and immunology, can provide important insights into the immunobiology and oncogenesis of lymphoid cancer.

### The non-Hodgkin lymphomas (NHDL) – Deciding on chemotherapy

J.M.A. Whitehouse

*CRC Wessex Medical Oncology Unit, Southampton General Hospital, Southampton SO9 4XY, UK.*

The clinical behaviour of patients with NHDL is indicative of different pathologies rather than a single disease type. The relationship between morphological characteristics and the natural history of broad sub-types supports the existence of distinct entities. A classification of the non-Hodgkin lymphomas based on morphological characteristics, having precise and reproducible clinical relevance has not yet emerged, but the National Cancer Institute Working Formulation offers a reasonable compromise for international use. Clinical studies have for some time identified variations in behaviour and therapeutic outcome in patients with apparently similar disease extent and histologies. Immunological studies and, more recently, the use of DNA probes have underlined the heterogeneity of some groups which are apparently well defined morphologically. It seems likely that further sub-categories of NHDL will eventually emerge based on the application of these approaches. Such precise definition may contribute to a better understanding of the natural history of different lymphomas, but will only be of clinical value if the sub divisions prove to have therapeutic relevance. The curability of some NHDL and incurability of others, have already been recognised. The stimulus to explore new approaches to therapy will come from the potential to influence the behaviour of newly identified sub groups. Factors influencing treatment strategy, and the current objectives of therapy will be reviewed.

### Treatment of human lymphoma with derivatives of anti-idiotype antibodies

G.T. Stevenson

*Lymphoma Research Unit, Tenovus Laboratory, General Hospital, Southampton SO9 4XY, UK.*

Lymphomas offer a wealth of differentiation antigens for exploitation either as tumour markers or as targets for therapeutic antibody. Preeminent antigens as regards specificity and characterisation are the idiotypes of surface Ig and the T-cell receptor. Therapeutic antibody can damage the tumour by delivering to it an exogenous toxic agent, or by recruiting the host's own cytotoxic agents such as complement and various cellular effectors. The latter strategy is the one that we have studied the more intensively.

We are using chimeric univalent derivatives of monoclonal antiidiotype antibodies to treat human B-cell lymphoma. The derivatives recruit host effectors efficiently, avoid rapid antigenic modulation, and have a good metabolic survival. Our current protocol provides them for intramuscular injection, which is vastly more convenient than intravenous infusion. Results, problems and future trends will be discussed.

### Abstracts of members' proferred papers

### Growth and morphology of a cell line from Jaagsiekte, a contagious lung tumour of sheep, can be manipulated *in vitro*

F.A. Jassim, J.M. Sharp, K.W. Angus & J. Menzies

*Moredun Research Institute, 408 Gilmerton Road, Edinburgh EH17 7JH, UK.*

Jaagsiekte is a contagious lung tumour of sheep in which two secretory epithelial cells in the lower respiratory tract are transformed. These cells are type II pneumocytes in the alveoli and the cells of Clara in the terminal bronchioles. A cell line (JS7) has been established from the lungs of sheep with jaagsiekte and has been propagated continuously *in vitro* for more than 140 passages. The cells possess properties of transformed cells such as growth in soft agar and athymic mice, yet retain many of the characteristic differentiated features of type II pneumocytes. The effect of bromohexine

HCl and prednisolone on the morphology and ultrastructure of JS7 cells was studied. Cells were cultured for up to six days in medium with various concentrations of the two chemicals. Cells treated with prednisolone lost their squamous epithelial shape and assumed a fusiform swirling appearance. Coincident with this change in morphology, they also lost the characteristic cytoplasmic lamellar bodies but not apical microvilli nor desmosomes. Cells treated with bromohexine HCl showed an increase in the number and size of lamellar bodies. These data indicate that differentiated features of this important pulmonary epithelial cell can be manipulated *in vitro*.

**Chemosentivity testing of human lung cancer cell lines using a colorimetric assay**

J. Carmichael, W.B. De Graff, J. Gamson, A.F. Gazdar, J.D. Minna & J.B. Mitchell

*Naval Med. Oncol. Branch, NCI and Naval Hospital, Bethesda, Maryland, USA.*

A colorimetric assay was used to determine the chemosensitivity profile of 30 human lung cancer cell lines. The assay is based on the cellular reduction of MTT, a tetrazolium salt, to a purple coloured formazan product which can be measured spectrophotometrically, with production of formazan proportional to viable cell number. Of 15 small cell lung cancer lines (SCLC) tested, 7 were derived from previously untreated patients (Un/T). The non-small cell lung cancer lines (NSCLC) comprised 4 adenocarcinoma, 3 large cell, 2 adenosquamous, 2 bronchioloalveolar, 2 squamous and 2 mesothelioma cell lines. Chemosensitivity was assessed by following continuous exposure to drugs using a 4 day assay, with the $ID_{50}$ defined as the dose of drug resulting in a 50% reduction in MTT formazan production. Representative $ID_{50}$ values are illustrated in the following table:

| | Un/T SCLC ($n=7$) | T-SCLC ($n=8$) | NSCLC ($n=15$) |
|---|---|---|---|
| Melphalan (μM) | 3.98 | 17.28 | 37.04 |
| *Cis*-platinum (μM) | 0.88 | 2.54 | 4.03 |
| Adriamycin (nM) | 26.28 | 167.24 | 205.40 |
| VP-16 (μM) | 1.24 | 10.26 | 31.44 |

For all 4 drugs, Un/T SCLC lines were more sensitive than T-SCLC lines, with the NSCLC lines least sensitive. These findings are in keeping with clinical experience using chemotherapy in these cell types. This colorimetric assay can be semi-automated and therefore offers a rapid, reproducible assay for the chemosensitivity testing of cell lines.

**A high molecular weight (MW) non-bombesin/gastrin releasing peptide (GRP) growth factor in small cell lung cancer (SCLC)**

J.L. Millar, V. Macaulay, G.P. Joshi & I.E. Smith

*Section of Medicine, Institute of Cancer Research, and Lung Unit, Royal Marsden Hospital, Sutton, Surrey, UK.*

Autocrine growth factor secretion may explain rapid proliferation in SCLC. Bombesin/GRP (MW 1620/2800) is produced by and mitogenic to 'classic' SCLC cultures, but is absent in the more rapidly growing 'variant' lines. We are studying a 'classic' SCLC line, HC12, which proliferates in serum-free medium, without specific supplements. HC12 conditioned medium (CM) was lyophilised, reconstituted in aqueous solution and dialysed against 0.9% saline across cellulose membrane (approx. MW cut-off 10–12 kDa). The final preparation (FD CM) combined no detectable bombesin-like immunoreactivity (BLI). We have developed a novel growth assay in which uptake of $^{14}$C-thymidine ($^{14}$C), $^{3}$H-uridine ($^{3}$H) and $^{75}$Selenomethionine ($^{75}$Se) indicate the rate of synthesis of DNA, RNA and protein respectively. Label uptake is linear with time in control HC12 cells growing in RPMI alone. Addition of FD CM stimulates incorporation of $^{14}$C (by 500% over control at 40 h), $^{3}$H (220%), and $^{75}$Se (130%); enhanced label uptake correlates with increase in cell number. The bombesin-homologous fragment of GRP (amino acids 14 to 27, at 5 μg ml$^{-1}$) caused less marked stimulation of uptake of $^{14}$C (210% at 40 h), $^{3}$H (108%) and $^{75}$Se (81%). Notably, the GRP effect on DNA synthesis was less than that reported by Weber *et al.*, 1985 (*J. Clin. Invest.*, **75**, 306). HC12 expresses BLI, but bombesin/GRP receptors may be down regulated in the presence of high MW growth factor/receptor interaction. HC12 FD CM also stimulates label uptake in another 'classic' SCLC line; we plan targetting experiments in other SCLC and non-SCLC lines, and hope to further characterise this putative growth factor.

**Cytotoxic drug targetting to lung cancer cells *in vitro* with a complex of low density lipoprotein (LDL)-daunomycin**

D.J. Kerr[1], S. Hynds[2], T.E. Wheldon[3], J. Shepherd[2] & S.B. Kaye[1]

[1]*Department of Medical Oncology and* [2] *Clinical Biochemistry, University of Glasgow;* [3]*Radiobiology Group, Belvidere Hospital, Glasgow, UK.*

High affinity LDL-receptors have been demonstrated on the cell surface of epidermal cervical carcinoma cells, glioma and P388 and human leukaemic cells *in vitro*. Using $^{125}$I-labelled LDL we have demonstrated high affinity saturable binding sites for LDL (Vmax = 125 cpm μg$^{-1}$ cell protein, Km = 40 μ ml$^{-1}$) on a human non-small cell lung tumour line (L-DAN). A stable LDL-daunomycin complex has been synthesised and its cytotoxicity in monolayer and spheroid, cellular uptake and metabolism has been compared to free daunomycin. The respective monolayer clonogenic $ID_{90}$s for LDL-daunomycin and free daunomycin are 1.0 μg ml$^{-1}$ and 1.2 μg ml$^{-1}$. Drug uptake studies were performed on monolayers, following exposure to drug at fixed concentration (5 μg ml$^{-1}$) for up to 2 h. Intracellular daunomycin and its metabolite daunomycinol were measured using HPLC. Daunomycin was extensively metabolised to the relatively inactive daunomycinol, so that by 2 h the alcohol predominated over the parent drug. Intracellular daunomycin levels were similar following exposure to both drugs, although total intracellular drug (daunomycin + daunomycinol) was 50% higher after free daunomycin, implying that LDL protects daunomycin from metabolism. L-DAN spheroid growth delay was measured following treatment with both free and LDL-bound daunomycin. LDL-daunomycin induced significantly longer growth delay at each concentration and appeared to increase the intra-spheroidal depth to which daunomycin diffused, as assessed by fluorescent microscopy. It is possible that the LDL-daunomycin complex increases daunomycin penetration in spheroids and enhances cytotoxicity.

**Determinants of prognosis in small cell lung cancer at presentation and in response to chemotherapy**

H. Earl[1], R.L. Souhami[1], C.M. Ash[1], S.G. Spiro[2], P.G. Harper[3], J.S. Tobias[1] & D.M. Geddes[4]

[1]*University College Hospital,* [2]*Brompton Hospital,* [3]*Guy's Hospital,* [4]*London Chest Hospital, UK.*

Clinical and biochemical parameters in patients treated in a randomised prospective trial of 370 patients were analysed for their influence on prognosis, using a multiple regression analysis. All of the prognostic information was given by initial performance, serum sodium, albumin, alkaline phosphatase and disease extent, all measured at presentation. Patients in whom all the biochemical factors were normal with performance status (Karnovsky) >70 and limited disease, had a greater than 15% chance of surviving 2 years. A separate analysis on a new data set of 640 patients has confirmed these findings and has shown a quantitative relationship between survival, performance status, and level of alkaline phosphatase. Almost all the long survivors in this new study had limited disease and no biochemical abnormality at presentation.

Further analyses have shown that the effect of adverse prognostic factors at presentation diminished with time. Detailed analyses of sequential data indicates that if treatment normalises adverse factors, the prognosis improves. These data are of significant help in deciding how long chemotherapy should continue especially in patients with an initially poor prognosis.

### Increased N-*myc* expression in human small cell carcinoma of the lung indicate poor prognosis

J. Bergh[1], E. Nöu[2] & K. Fina[3]

[1]*Department of Oncology,* [2]*Department of Lung Medicine,* [3]*Department of Immunology, University of Uppsala, S-751 85 Uppsala, Sweden.*

Human small cell carcinoma of the lung (SCCL) is characterized by an initial prompt response to radio- and chemotherapy. Unfortunately, ~90% of the patients relapse within 5 years with clones of SCCL, which are resistant to therapy. SCCL cell lines have previously been demonstrated, in certain instances, to have amplified and increased expression of the proto-oncogenes c- and *N-myc*. SCCL cell lines with increased levels of c-*myc*, mostly have a more rapid growth and also a decreased expression of some neuroendocrine markers for SCCL *vs.* non-SCCL (squamous-, adeno- and large cell carcinomas of the lung). In this study we have examined biopsies from 15 untreated patients with SCCL using $^{35}$-S-labelled RNA probes against N-*myc*. Deparaffinized sections were used after proteinase K and acetic anhydride- glycine treatment with a 1.7 Kb EcoRI-Bgl11 genomic fragment of the third exon of the human N-*myc*. A primary lung-adenocarcinoma biopsy was used as positive control, while this tumour previously has been demonstrated to contain N-*myc* amplification using Southern blots. To evaluate the results of the SCCL biopsies the number of grains was calculated. Six of 7 patients with complete responses and a median survival time of 22 months (range 9–64 months) were negative for N-*myc* expression. The 6 patients with N-*myc* positive SCCL biopsies had, together with 2 negative patients, only partial- or no responses; with a median survival of 13 months (range 6–16 months).

### Intermittent chemo/radiotherapy in small cell lung cancer: A novel approach to combined modality therapy

M.H. Cullen, N.S. Stuart, C.M. Woodroffe, R.J. Grieve[1], A.D. Chetiyawardana, T.J. Priestman, D. Spooner & G.H. Benfield

*Department of Radiotherapy and Oncology, Queen Elizabeth Hospital, Birmingham,* [1]*Department of Radiotherapy, Coventry and Warwickshire Hospital, UK.*

The role of thoracic radiotherapy (TRT) in small cell cancer is controversial even in limited disease (LD). Concurrent chemotherapy (CT) and TRT may confer benefit, but at the expense of substantial local toxicity. In this study TRT is used as a localized cytotoxic agent given intermittently the day following i.v. CT.

Forty-eight patients are currently on study. Eleven patients received VACR (d1: vincristine 1 mgm$^{-2}$, adriamycin 40 mgm$^{-2}$, cyclophosphamide 1000 mgm$^{-2}$ i.v.; d2: TRT 400 cGY-q21d x 6). Thirty-seven patients received RAVE (d1: vincristine 1 mgm$^{-2}$, adriamycin 40 mgm$^{-2}$, etoposide 120 mgm$^{-2}$ i.v.; d2: etoposide 250 mgm$^{-2}$ po, TRT 400 cGy (17 patients), 500 cGy (20 patients)-q21d x 6). Thirty-six patients have LD, 12 extensive disease (ED). Forty-seven are evaluable for response:

| *Stage* | *Eval. for response* | *CR (X-ray)* | *CRb−* | *CRb+* | *CRbo* | *PR* | *F* |
|---|---|---|---|---|---|---|---|
| LD | 35 | 23 | 9 | 2 | 12 | 8 | 4 |
| ED | 12 | 7 | 4 | 1 | 2 | 1 | 4 |

*b−* = bronchoscopy negative; *b+* = bronchoscopy positive; *bo* = bronchoscopy refused.

Of 30 CR patients, 22 have relapsed. Only 6 of these have occurred within the TRT field. Sixteen have occurred at distant sites with no local failure. Three of these were in the CNS.

The median survival of LD patients is 65.5 weeks and 29 weeks for ED. Four patients are alive and disease free at 95+–171+ weeks.

The toxicity of concurrent therapy has been minimal. Two patients have had symptomatic oesophagitis, both transient. Radiation pulmonary fibrosis was present on X-ray at 6 months in 11/14 evaluable patients having 6 x 500 cGy fractions, but no other late effects of RT have occurred. Intermittent chemo/radiotherapy may induce high response rates in the chest with little symptomatic local toxicity and few in-field recurrences.

### *In vitro* cross-resistance patterns of anthracyclines in human non-small cell lung cancer cell lines with differing inherent resistance to adriamycin

S. Merry, E.R. Courtney, C. McCormick, S.B. Kaye & R.I. Freshney

*CRC Department of Medical Oncology, University of Glasgow, Glasgow, UK.*

In order to assess the potential lack of cross-resistance with adriamycin (ADR) of 5 novel anthracycline analogues, studies have been performed with 2 human non-small cell lung cancer cell lines (A549 and SK-MES-1) of differing inherent sensitivity to ADR. The 5 compounds are idarubicin (IDA), 4′-deoxy-4′-iododoxorubicin (JODO) both supplied by Farmitalia; a 9-methyl derivative (Ro31/1215) supplied by Roche; a cyanomorpholino derivative (MRA-CN) supplied by SRI International, and a 7-hydroxyanthra-pyrazole (CI941) supplied by Warner Lambert. Cells of the 2 cell lines were seeded onto microtitre plates and, after 96 h, were exposed to drug for a further 72 h (with drug replacement at 24 and 48 h) followed by a recovery period of 120 h. Viability at the end of the assay was assessed as tritiated leucine incorporation. Results expressed as $ID_{50}$ values ($\times 10^{-9}$ M) were:

| | *A549* | *SK-MES-1* | *RATIO* |
|---|---|---|---|
| ADR | 210 | 18 | 12 |
| IDA | 0.26 | 7.3 | 0.036 |
| JODO | 0.038 | 6.0 | 0.0063 |
| Ro-31/1215 | 0.89 | 13 | 0.0068 |
| MRA-CN | 0.18 | 0.077 | 2.3 |
| CI941 | 15 | 2.0 | 7.5 |

These results suggest a relative lack of cross-resistance for all these compounds except CI941, and (in our model) JODO and Ro31/1215 look most promising in this respect.

### Cyclosporins overcome multi-drug resistance in human lung cancer and mouse tumour cell lines

P.R. Twentyman, N.E. Fox & D.J.G. White[1]

*MRC Clinical Oncology and Radiotherapeutics Unit and [1]University of Cambridge, Department of Surgery, Hills Road, Cambridge, UK.*

We have developed multi-drug resistant variants of the NCI-H69 human small cell lung cancer and the EMT6 mouse tumour cell lines. These variants show high degrees of resistance to adriamycin (ADM), vincristine (VCR) and colchicine. Many different chemical compounds are currently being studied with regard to their ability to remove this resistance. Growth inhibition during continuous drug exposure is used as the cytotoxic drug response endpoint and resistance factor (RF) is the ratio of drug doses to produce 50% inhibition in the resistant and parent lines.

Both parent cell lines were more sensitive to the immunosuppressive drug cyclosporin A (CYA) than were their multidrug resistant variants. For ADM, the RF for the variant lung cancer line is around 100. CYA ($5\mu g\,ml^{-1}$) made little difference to the ADM sensitivity of the parent line but considerably sensitised the resistant line, thereby reducing the RF to around 3. The magnitude of the effect was reduced with reducing dose of CYA, but some sensitisation was still observed at $0.5\mu g\,ml^{-1}$. Similarly the RF for VCR could be reduced from 1,000 to 10 by $5\mu g\,ml^{-1}$ of CYA. This resulted from a 300-fold sensitisation of the resistant line compared with a 3-fold sensitisation of the parent line. Cyclosporin C and cyclosporin G, analogues possessing immunosuppressive properties, each showed similar activity to CYA at a dose of $5\mu g\,ml^{-1}$. The biologically inert analogue cyclosporin H, however, had little or no activity at $10\mu g\,ml^{-1}$. Qualitatively similar results were obtained using the drug resistant variant of the EMT6 cell line.

These studies suggest that cyclosporins, at clinically achievable doses, may have a role as specific sensitisers of chemotherapy-resistant tumours.

### The radiation responses of human lung tumour lines of different histological types

G. Duchesne, J. Peacock & G. Steel

*Institute of Cancer Research, Sutton, Surrey SM2 5PX, UK.*

The acute *in vitro* responses to radiation of a panel of human lung carcinoma lines, representing 3 of the common histological types, were determined using a soft agar clonogenic assay. (A squamous carcinoma failed to clone using this technique). The survival data were analysed according to the multitarget and linear quadratic models, and the surviving fraction at 2 Gy ($SF_2$). The mean Do of the classical small cell carcinomas ($1.26\pm0.11$) was significantly lower than that of the other cell types ($1.63\pm0.09$, $P<0.01$). Significant differences in $\alpha$, representing the initial slope of the cell survival curve, were also found between the lines: the mean $\alpha$ of the lines with a large cell component ($0.19\pm0.03$) was lower than that of the small cell lines ($0.57\pm0.06$, $P<0.01$) and the adenocarcinomas ($0.38\pm0.06$, $P<0.05$), suggesting lower intrinsic sensitivity in the large cell lines. This was confirmed by comparison of $SF_2$: large-cell $0.70\pm0.05$, small-cell $0.34\pm0.03$, $P<0.01$, adenocarcinoma $0.43\pm0.13$, $P=0.05$. No differences in repair of sublethal or potentially lethal damage were observed. The results suggest that the clinical observations of resistance in large cell tumours is due to intrinsic cellular resistance. Improved therapeutic ratios may be obtained by the use of hyperfractionation in the management of small cell carcinomas and adenocarcinomas.

### Control of growth on non-small cell lung cancer by dexamethasone

J. McLean & R.I. Freshney

*Department of Medical Oncology, University of Glasgow, 1 Horselethill Road, Glasgow G12 9LX, UK.*

*In vitro* experiments have shown that glucocorticoids are cytostatic for glioma and both small cell and non-small cell lung carcinoma cells. The effect is most pronounced in late log and plateau phase cultures implying cell–cell contact may be important. Cell surface modifications are also induced by dexamethasone (DX) and methyl prednisolone inducing a reduction in hyaluronic acid released into the medium correlating with cytostasis and, possibly, enhanced cell adhesion. DX also reduces plasminogen activator activity and the release of mitogenic factors for endothelium by tumour cells *in vitro*, while enhancing surfactant production in A549 alveolar carcinoma cells. This implies a shift towards a less malignant phenotype. *In vivo* studies have shown reduction in tumour growth in xenografts in nude mice with increased central necrosis possibly due to inhibition of angiogenesis. Treatment of NCI-H125 which did not give a typical cytostatic or phenotypic response *in vitro*, gave no inhibition of growth in xenografts implying a cellular rather than systemic effect of the steroid.

### DNA ploidy in pulminary carcinoid tumours

D.J. Jones[1], P.S. Hasleton[2] & M. Moore[1]

*[1]Paterson Institute for Cancer Research, Christie Hospital & Holt Radium Institute, Manchester and [2]Department of Pathology, Wythenshawe Hospital, Manchester, UK.*

Carcinoid tumours account for less than 10% of bronchopulmonary neoplasms. They exhibit a spectrum of histological features and clinical behaviour, varying from histologically typical endobronchial tumours to malignant metastasizing neuroendocrine carcinomas. DNA ploidy was assessed by flow cytometry on paraffin embedded material obtained from 53 patients presenting between 1961 and 1985, according to the method of Hedley *et al.*, *J. Histochem. Cytochem.* **31**, 1333, 1983), using a Coulter EPICS flow cytometer. DNA ploidy was correlated with the following conventional pathological features (a) type – typical/atypical, (b) growth pattern – insular/trabecular/glandular/undifferentiated, (c) vascular invasion (d) lymphatic invasion, (e) nuclear pleomorphism (f) necrosis (g) lymph node involvement. Statistical analysis was by Chi squared test. 26/53 were DNA aneuploid. DNA aneuploidy rates for pathological subgroups were as follows: 9/28 typical, 17/25 atypical ($P<0.02$); 11/32 insular, trabecular and glandular combined; 15/21 undifferentiated ($P<0.02$); 21/26 with, 5/27 without nuclear pleomorphism ($P<0.001$); 13/18 with, 13/35 without necrosis ($P<0.05$); 11/13 involved lymph nodes; 12/36 uninvolved lymph nodes ($P<0.01$). There was no significant correlation between DNA aneuploidy and vascular or lymphatic invasion. In conclusion, DNA aneuploidy is a feature of pulmonary carcinoid tumours and is associated with other pathological features of increasing malignancy. DNA ploidy status is not helpful in discriminating carcinoids from other lung tumours as previously suggested (Blöndal *et al.*, *Eur. J. Respir, Dis.*, **64**, 298, 1983). The presence of a detectable aneuploid population is not a prerequisite for malignant behaviour and aneuploidy does not invariably confer a malignant phenotype. Subchromosomal structural changes and altered gene expression are probably as important as gross numerical aberrations.

### Measurement of intracellular carbamoylating activity of chloroethylnitrosoureas in intact human small cell carcinoma (NCI-H69) and murine mammary tumour (EMT6) cells using flow cytoenzymology

C. Dive, P. Workman & J. Watson

*MRC Clinical Oncology and Radiotherapeutics Unit, MRC Centre, Hills Road, Cambridge, UK.*

As well as yielding DNA-interacting alkylating fragments, chloroethylnitrosoureas decompose under physiological conditions to generate isocyanates which carbamoylate intracellular proteins. Carbamoylation has been implicated in the inhibition of DNA repair and RNA processing, enhancement of radiation cytotoxicity, and degree of myelosuppression. We have developed a novel method of measuring protein carbamoylation based on flow cytoenzymology. The major advantage over previous techniques is that the reaction is measured in intact cells, so that membrane penetration is considered as well as biochemical reactivity. The assay measures inhibition of esterases using the substrate fluorescein diacetate, which on conversion to fluorescein accumulates in the cell reflecting enzyme activity. Cells were preincubated with drug for 1 hr before addition of substrate. Fluorescence was measured with time using the Cambridge flow cytometer, cell population progress curves constructed and the concentration of drug required to produce 50% inhibition ($I_{50}$) was calculated for a wide range of structural analogues. Results for the human and murine cells were very similar, and the drugs could be classified into high, intermediate and low inhibitory potency. BCNU, ACNU and chlorozotocin are examples of each category, with $I_{50}$ values of $7\times10^{-4}$ M, $1\times10^{-2}$ M and $\gg10^{-2}$ M respectively. Though broadly in agreement with results of previous methods, important differences were identified, e.g. GANU exhibited weak activity in this assay, partly due to poor membrane permeability. This assay can now be used to study the cellular pharmacology of novel chloroethylnitrosoureas under development.

### Immunohistochemical staining patterns of small cell cancer of the lung (SCCL) – Relationship to prognosis

S.G. Allan, F.G. Hay & R.C.F. Leonard

*Imperial Cancer Research Fund Medical Oncology Unit, University Department of Clinical Oncology, Western General Hospital, Edinburgh, EH4 2XU, UK.*

SCCL remains a highly lethal disease despite good response rates achieved by chemotherapy. Potentially the linking of clinical events to cellular behaviour could provide insight into tumour response and prognosis. Using a panel of monoclonal antibodies (MoAbs) we have examined tumour cell antigen expression in a series of 38 tissue sections from patients with SCCL in whom clinical information was available. Thirty-eight paraffin-embedded blocks fixed in 10% buffered formalin (31 primary lesions, 7 metastatic) were cut as 5 $\mu$m sections. After dewaxing and rehydration, endogenous peroxidase activity was blocked and sections were trypsinised prior to incubation with MoAbs. HMFG1, HMFG2 and CAM 5.2 were used as undiluted supernatants. Anti-leu 7, B5, bombesin, EFG-RF4, Mo2, 534F8 and c-*myc* were used at optimal dilutions. Thereafter a standard 3 step PAP technique was applied and the end product developed using $DAB/H_2O_2$ with haematoxylin counter staining. In the 31 primary lesions positive staining (10% or more) was achieved in 17/31 HMFG2, 10/15 HFMG1, 27/29 B5, 15/31 534F8, 2/31 EGF/RF4, 5/18 Mo2, Leu-7 3/28, bombesin 0/18, c-*myc* 0/22. A positive inverse relationship with survival was demonstrated for HMFG2 staining – mean 8.1 mo for HMFG2 positive *vs.* 15.2 mo for HMFG2 negative ($p<0.05$, Mann–Whitney). No relationship to survival was shown for other antibodies. In the few samples expressing F4 positivity survival was very short. Further studies of HMFG2 and its use as a marker in SCCL are indicated.

### Oral ifosfamide and etoposide for small cell lung cancer patients

M. Lind[1], N. Thatcher[1], T. Cerny[1] & K. Carroll[2]

[1]*CRC Department of Medical Oncology, Christie Hospital & Holt Radium Institute and* [2]*Wythenshawe Hospital, Manchester, UK.*

In elderly and poor prognosis patients with small cell lung cancer simple treatments with rapid responses and rapid improvement of symptoms with low toxicity are desirable. The response rate for i.v. ifosfamide and etoposide is 63% for extensive disease and 90% for limited disease. The bioavailability for oral ifosfamide is 100%.

We have treated 45 patients with SCLC with ifosfamide (2 g p.o. days 1–3 with equidose mesna) and oral etoposide 100 mg days 1–8 repeated every 3–4 weeks for a maximum of 6 courses.

There were 15 CRs and 22 PRs giving an overall response rate of 92%. There was only 1 treatment related death (septicaemia). There were no cases of cystitis and the incidence of severe encephalopathy 8%.

### Small cell lung cancer: A phase 2 evaluation of *r*-interferon-$\gamma$(immuneron) in 12 previously untreated patients

H. Newman & N.M. Bleehen

*MRC Clinical Oncology and Radiotherapeutics Unit, MRC Centre, Hills Road, Cambridge, UK.*

Although up to 80% of patients with small cell lung cancer will respond to chemotherapy initially, survival at 2 years is only 15%. Alpha interferons have not proved effective in this disease, and have shown significant toxicity (Jones D.H. *et al.*, *Br. J. Cancer*, **47**, 361, 1983). Gamma interferons differ from the $\alpha$-molecule, notably possessing greater cytotoxic and immunomodulatory activity. Immuneron (*r*-IFN-$\gamma$) has been shown to inhibit the growth of small cell lines *in vitro* (Twentyman, P.R. *et al.*, *Br. J. Cancer*, **52**, 21, 1985). This agent was therefore assessed under optimal conditions in previously untreated patients. A dose of 1 mg m$^{-2}$ day$^{-1}$ for 5 days continuously was followed by a maintenance dose of 0.5 mg m$^{-2}$ thrice weekly, being given i.v. over 30 min. The trial was stopped after 12 of the 14 projected patients had been treated, because of poor responses. Four patients are considered non-evaluable for response; one was withdrawn because of hypotension, one developed spinal cord compression, and two died from intercurrent illness not thought to be Immuneron related. Of the remaining 8 patients 4 had stable disease for 1 month, and 4 had progressive disease within 3 weeks on therapy. Toxicity consisted mainly of pyrexia (11/12) and malaise (10/12). The lowest total WBC was 2.0, and transient rise in

SGPT at the end of week 1 was seen in 9/12 patients. Paired serum for *E. coli* and *r*-IFN-$\gamma$ antibodies were taken and results will be presented. Ten patients subsequently received chemotherapy; there were 7 responses, 3 being complete. The mean duration of response was 8 months. It is concluded that Immuneron has no clinically significant single agent activity in small cell lung cancer.

**Neuron specific enolase and CEA compared in small cell lung cancer**

E.H. Cooper[1], L. Jørgensen[2], H. Hansen[2] & M.D. Peake[3]

[1]*Unit for Cancer Research, University of Leeds, Leeds LS2 9NL;* [2]*Finsen Institute, 2100 Copenhagen and* [3] *Pontefract General Infirmary, Pontefract WF8 1PL, UK.*

The serum levels of neuron specific enolase (NSE) and carcinoembryonic antigen (CEA) were measured in 98 patients with SCLC before treatment. In patients with limited disease 37/57 (64%) had a raised NSE $>12.5\,\mathrm{ng\,ml^{-1}}$ and 19/57 (33%) had a CEA $>6\,\mathrm{ng\,ml^{-1}}$, in 29/57 (51%), the CEA was $>3\,\mathrm{ng\,ml^{-1}}$. In extensive disease 33/41 (80%) had a raised NSE, and 18/41 (44%) a CEA $>3\,\mathrm{ng\,ml^{-1}}$. The combination of an NSE $>12.5\,\mathrm{ng\,ml^{-1}}$ and/or CEA $>6\,\mathrm{ng\,ml^{-1}}$ occurred in 70/98 (71%) patients at presentation. Levels of NSE $>25\,\mathrm{ng\,ml^{-1}}$ are highly suspicious of SCLC but at this level the test would only detect 46% of the patients in our series. With the exception of 5 non-responders, chemotherapy was associated with the NSE falling rapidly to reach $4–10\,\mathrm{ng\,ml^{-1}}$ after one or two courses. Partial and complete responses could not be distinguished by their NSE plateau levels. In 26 incidences of systemic progressive disease a rising NSE was observed in 23 (88%) of these incidences; a rising CEA was observed in 40%. Isolated metastases in the central nervous system did not produce a rise of NSE. Serum NSE can provide a monitor for patients with SCLC receiving chemotherapy, and provides evidence of progression during chemotherapy or after treatment has been completed.

**Antibody-guided targetting of non-small lung cancer using radiolabelled HMFG1 (Fab′)2 fragments**

H. Kalofonos, N. Courtney-Luck, J.P. Lavender, G. Hooker, G. Robinson, D. Snook, J. Taylor-Papadimitriou[1] & A.A. Epenetos

*Royal Postgraduate Medical School, Ducane Road, London, W12 0HS and Imperial Cancer Research Fund, Lincoln's Inn Fields, London, WC2 3PX, UK.*

(Fab′)2 fragments of tumour associated monoclonal antibody HMFG1 were prepared and radiolabelled with indium 111-In. Radiolabelled antibody was shown to be stable both *in vitro* and *in vivo* and there was no significant loss of immunoreactivity. Ten patients with non-small primary and metastatic lung cancer (NSCC) were studied by radio-immunoscintigraphy after i.v. administration of 111-In labelled (Fab′)2 HMFG1. Successful localisation was observed in all patients with no significant uptake in any normal tissue, except for liver (accumulating $\sim$20% of injected amount). A new radiolabelled 90 Yttrium has been chelated to HMFG1 (Fab′)2 fragments for potential antibody guided therapy of lung cancer. Following the procedure, there was no loss of antibody immunoreactivity. The radiolabelled antibody was found to be stable both *in vitro* and *in vivo* as studied in pre-clinical trials. We conclude that 111-In labelled HMFG1 (Fab′)2 fragments are an important new development in the radio-immunolocalisation of NSCC lung cancer and that 90-Y labelled antibody is potentially suitable for therapeutic use.

**Tumour marker measurements in spermatic vein blood of testicular cancer patients**

P.A. Light[1], & C.J. Tyrrell[2]

[1]*Radiotherapy and Oncology Centre, Bristol BS2 8ED and* [2]*Department of Radiotherapy, Plymouth General Hospital, Plymouth PL4 7JJ, UK.*

Alphafoetoprotein (AFP) and/or chorionic gonadotrophin (hCG) are present in the blood of many patients with testicular cancer, and the estimation of these markers pre- and post-operatively is now of established value in the management of this group of patients. A recent study of marker concentrations in spermatic vein blood (s.v.b., taken at the time of orchidectomy) has shown that these measurements may be of particular value in patients whose tumours produce only small amounts of marker(s), such that, although measurable in s.v.b. draining directly from the tumour, they fall below the level of detection after dilution in the peripheral circulation. Further studies have shown that the ratio of marker concentration between s.v.b. and peripheral blood (p.b.) may vary greatly between patients. The biggest differences were seen in patients in whom disease was subsequently found to be Stage 1 (i.e. disease confined to the testis), and in this group s.v.b. marker values were higher or very much higher than p.b. values. However, in patients who were subsequently found to have widespread or bulky metastatic disease, the s.v.b. marker values were only slightly greater than p.b. values or, in some cases, not significantly different. Assessment of such marker concentration ratios may assist in the early identification of metastatic disease.

**Expression of the c-*myc* oncogene product in gastric cancer and associated 'pre-malignant' lesions**

F. Macdonald, W.H. Allum, H. Stokes & B. Russell

*Surgical Immunology Unit, Queen Elizabeth Hospital, Edgbaston, Birmingham, UK.*

Clearly defined pre-malignant changes have not been identified in gastric cancer. However, both atrophic gastritis and intestinal metaplasia have been found in association with cancers. Dysplastic lesions have also been studied to determine their involvement with this disease. In an attempt to identify a marker which may indicate those conditions likely to develop into frank malignancy, we have examined the role played by oncogenes in the development of this disease. Tissues from patients with inflammatory, 'pre-malignant' and malignant conditions of the stomach as well as normals have been examined in an immunohistochemical test with an antibody to the c-*myc* oncogene protein.

Biopsies from 130 patients entered into a trial for the detection of early gastric cancer were examined. Strong expression of the c-*myc* protein was detected in 54% of patients with intestinal metaplasia, 66% of those with atrophic gastritis and 60% of those with dysplasia compared with only 10% of patients with superficial gastritis. Intense staining was seen in 7/10 patients who had atrophic gastritis in association with incomplete intestinal metaplasia – a condition linked with 'intestinal' cancers of the stomach. Staining in the 96 cancers tested was less intense but was found in 42% of those belonging to the 'intestinal' group compared with 23% of the 'diffuse' group. A similar pattern was observed in autologous lymph node metastases. Sequential biopsies obtained from two patients who developed early gastric cancer, following an initial diagnosis of intestinal metaplasia/atrophic gastritis, showed strong expression of c-*myc* in early biopsies. These results suggest that c-*myc* may be a useful marker to identify those lesions which will develop into frank malignancy.

**Radiotherapy employing three fractions each day over a continuous period of 12 days**

M.I. Saunders & S. Dische

*Marie Curie Research Wing, Regional Radiotherapy and Oncology Centre, Mount Vernon Hospital, Northwood, Middlesex HA6 2RN, UK.*

Biological evidence suggests that in the radiotherapy of human tumours regrowth between fractions is much more important as a cause for failure to control than was considered formerly. Accelerated fractionation using multiple treatments in one day should lead to a reduction in time for regrowth between fractions. Most workers who have used accelerated fractionation have not been able to complete treatment to a satisfactory total dose without introducing a split-course schedule. It follows, however, that with accelerated fractionation a pause in treatment during a rest period and/or during a week-end is likely to negate the benefit that may be obtained.

In order to avoid such interruptions a scheme of radiotherapy has been planned which gives a total tumour dose of 50.4 Gy in 36 fractions given three times a day over 12 consecutive days. In a pilot study of 39 patients 23 have shown advanced bronchial carcinoma. The predicted acute reactions in the oesophagus have not been excessive and no other immediate problems have developed. So far late changes appear no greater, and perhaps are less evident, than with conventional treatment, but the follow up time remains short. Immediate tumour responses are very promising, complete regression being observed in 40% to be compared with 14% in a previous comparable series. The good tolerance has now allowed an increase in the total dose to 54 Gy.

**Psychosocial sequelae of mastectomy *v*. breast conservation**

L.J. Fallowfield[1], M. Baum[1] & G.P. Maguire[2]

[1]*CRC Trials Centre, Department of Surgery, King's College Hospital, London SE5 and* [2]*Department of Psychiatry, University Hospital of S. Manchester, Manchester M20 8LR, UK.*

High levels of psychiatric morbidity are well-known sequelae of a diagnosis of breast cancer and its treatment by mastectomy. This, together with survival data accumulating from studies comparing mastectomy with more conservative surgery, has led many surgeons to offer breast conservation as primary treatment. There is to date no satisfactory empirical evidence supporting the intuitive assumption that local excision and radiotherapy protects women from the anxiety and depression experienced by patients who undergo mastectomy. The CRC's Breast Conservation Trial provided a unique opportunity to examine this issue. We present psychological data from 101 women randomised to either lumpectomy or mastectomy. The levels of psychiatric morbidity as measured by a semi-structured interview and 2 self-report questionnaires were similar in both treatment groups. Approximately 15 months post-operatively, 21% of the mastectomy patients were depressed and 26% were anxious. Amongst the lumpectomy patients 27% were depressed and 31% experiencing anxiety. Furthermore, over one third of patients in both groups reported a lack of sexual interest. These unexpected results are discussed, together with their implications for clinical practice and counselling services.

**Oral CGP 32349 (4-hydroxyandrostenedione) has antitumour activity in breast cancer**

D. Cunningham[1], T.J. Powles[2], M. Dowsett[3], H.T. Ford[1], J.-C. Gazet[1], G. Hutchison[1], A.M. Brodie[4] & R.C. Coombes[1,2,5]

[1]*St. George's Hospital, London SW17;* [2]*Royal Marsden Hospital, Surrey;* [3]*Chelsea Hospital for Women, London SW3;* [4]*University of Maryland School of Medicine, Baltimore USA and* [5]*Ludwig Institute for Cancer Research (London Branch) St. George's Hospital Medical School, London SW17, UK.*

We have previously shown that 4-hydroxyandrostenedione (4-OHA), a potent aromatase inhibitor has significant antitumour activity when administered parenterally to post-menopausal women with breast cancer. This is the first report of the use of 4-OHA administered orally.

Thirty-one female patients, mean age 65.7 yrs (range 40–82 yrs) with locally advanced or inoperable breast cancer were treated with 4-OHA given orally at a dose of 500 mg daily. All were postmenopausal. Seventeen (54%) had been given previous endocrine therapy with 1 agent, 6 (19%) had received 2 agents and 3 (10%) had received 3 agents with an overall response rate of 60%. Response to 4-OHA was as follows. Eight (26%) had a partial response (PR) with a median duration >10 months; 6 remain in remission. Four (13%) patients had stable disease (SD), 11 (36%) had progressive disease (PD) and 8 (25%) were not evaluable. Ten patients had ER positive tumours and 5 (50%) responded (PR+SD). One of 9 patients with ER negative tumours responded and 6 (50%) of 12 patients with ER unknown tumours responded (PR+SD). In 16 of the 31 patients serial plasma oestradiols were measured and were lowered to $53 \pm 8\%$ of baseline levels within 7 days of commencing 4-OHA. Thirty patients were evaluable for toxicity. Twenty-seven (90%) experienced no side effects; a patient had an erythematous skin rash and 1 patient had facial swelling. In 1 patient treatment was discontinued due to leucopaenia (WBC = 2.5).

Oral 4-OHA is a new treatment for post menopausal women with breast cancer which has few side effects.

**Repeat bone marrow aspirates in patients with primary breast cancer**

J.L. Mansi, U. Berger, T. McDonnell & R.C. Coombes

*Ludwig Institute for Cancer Research (London Branch), St. George's Hospital, London SW17 0RE, UK.*

A major problem in preventing relapse in breast cancer patients is selecting the appropriate adjuvant therapy and knowing for how long to treat. In order to try and address this problem we have sampled marrow for micrometastases before and after adjuvant therapy. Tumour cells were found in the bone marrow of 26.4% (81/307) of patients with primary breast cancer when multiple aspirates are taken at the time of initial surgery and stained with an antiserum to epithelial membrane antigen (EMA). We have now repeated multiple aspirates in 75 patients at a mean time of 15 months after surgery. In 36 patients who had not received adjuvant therapy, 15 had micrometastases initially and 9 remained positive, and of 21 that were negative for tumour cells initially 17 remained negative. In contrast, of the 39 patients who have received adjuvant therapy 9/12 (75%)

became negative and 7/27 (26%) who were initially negative became positive during therapy. These results indicate that, whilst a minority of patients who do not receive adjuvant therapy become negative for micrometastases, 75% of patients who receive treatment become negative indicating possible effectiveness of therapy. We now intend to repeat the study in these patients at annual intervals to determine how long they eradicate micrometastases.

### High dose BCNU primary chemotherapy with full dose radiotherapy for astrocytoma grade IV

E. Mbidde, P. Selby, P. Workman, A. Whitton, T.J. McElwain & H.J.G. Bloom

*Departments of Medicine and Radiotherapy, Royal Marsden Hospital, Sutton and MRC Clinical Oncology and Radiotherapeutics Unit, Cambridge, UK.*

We are evaluating the use of high dose BCNU (800 mg–1 g m$^{-2}$) with autologous bone marrow grafting as the first post-surgical treatment of patients with astrocytoma grade IV. It is followed by full dose radiotherapy. Detailed pharmacokinetic studies using reverse phrase HPLC indicate that peak plasma concentrations range from 8.5–15.9 $\mu$g ml$^{-1}$ in the 5 patients studied. Rapid biphasic elimination occurs in the first 12 h, with concentrations at that time ranging from 0.03–0.056 $\mu$g ml$^{-1}$ with some evidence of a third phase. Returning the bone marrow graft at 24–30 h allowed successful reconstitution.

Seventeen patients have been treated on this programme. Median follow up 1 year (range 3–27 months). Nine patients remain alive, with 2 year actuarial probability of survival of 55%, comparing favourably with historical experience. Leucopenia and thrombocytopaenia were of short duration, but there were 3 cases of pneumonitis, 2 of which were fatal, and 1 fatal hepatic toxicity. This approach to high grade glioma deserves further evaluation in a controlled trial.

### Is high dose melphalan (HDM) of value in treatment of advanced neuroblastoma (AN)? Preliminary results of a randomized trial by the European Neuroblastoma Study Group (ENSG)

J. Pritchard[1], S. Germond[1], D. Jones[2], J. de Kraker[4] & S. Love[3]

[1]*Hospital for Sick Children, London WC1N 3JH;* [2]*St. George's Hospital Medical School;* [3]*ICRF, London and* [4]*Emmakinderziekenhaus, Amsterdam for the ENSG.*

The value of high dose consolidation chemotherapy has not been tested by randomized study in any paediatric solid tumour. In 1/82, the ENSG started a randomized Trial to test the value of HDM in children with AN. Up to 3/85, 140 patients (pts) over 6 mo. of age with 'Evans' stage 3 or 4 disease, consecutively referred to 18 centres, were given 3–4 weekly OPEC induction therapy – day (d) 1: vincristine 1.5 mg m$^{-2}$, cyclophosphamide 600 mg m$^{-2}$, d2: cisplatin 60 mg m$^{-2}$. d4: VM26 150 mg m$^{-2}$. Surgical removal of primary tumour was carried out before or during OPEC. There was no radiotherapy. Ninety-five pts (68) achieved complete (CR = disappearance of all evidence of tumour) or good partial response (GPR = disappearance of all evidence of secondary deposits and shrinkage of primary by > 50% in 3 dimensions) and were eligible for randomization after stratification for stage and centre. Because of non-compliance by some parents and physicians only 65 pts were randomized, 32 to HDM (180 mg m$^{-2}$) with autologous bone marrow rescue) and 33 to no further treatment (NFT). There were 2 HDM-related deaths from sepsis/neutropenia but life-table analysis at 9/85 showed that pts in the HDM group had better disease-free survival ($P = 0.03$ logrank) and survival ($P = 0.03$) than those in the NFT group. Though more follow-up is needed to ascertain eventual outcome we conclude that HDM/ABM is of benefit to children with AN who have responded to OPEC and surgery.

### Cardiotoxicity of mitozantrone assessed by stress and resting nuclear ventriculography

J. Cassidy[1], M.V. Merrick[2], J.F. Smyth[1] & R.C.F. Leonard[1]

[1]*University Department of Clinical Oncology and* [2]*Department of Nuclear Medicine, Western General Hospital, Edinburgh EH4 2XU, UK.*

Twenty-eight patients with advanced breast cancer who had not previously received cytotoxic therapy or mediastinal radiotherapy were randomised to treatment with combination chemotherapy using vincristine 1.4 mg m$^{-2}$, doxorubicin 50 mg m$^{-2}$ and prednisolone 40 mg orally days 1–5 (VAP), or vincristine 1.4 mg m$^{-2}$, mitozantrone 14 mg m$^{-2}$, prednisolone 40 mg orally days 1–5 (VMP) every 21 days. Before, during and after cessation of treatment radionuclide assessment of ventricular performance was obtained at rest, in response to cold pressor stress and on recovery from stress. Six of 14 patients (43%) in the VAP group, and 6 of 14 patients (43%) in the VMP group developed abnormalities of left ventricular ejection fraction. One patient receiving VMP developed congestive cardiac failure. Mitozantrone is an active agent in the treatment of advanced breast cancer but can produce cardiotoxicity. In this middle-aged population with no other risk factors the instance was similar to that seen with doxorubicin. Cardiotoxicity occurred over a wide range of cumulative doses (25–112 mg m$^{-2}$). Further investigation is required to determine the nature and prognosis of this condition.

### Long-term side-effects of chemotherapy for testicular cancer

N.S.A. Stuart, C.M. Woodroffe, R. Grundy & M.H. Cullen

*Queen Elizabeth Hospital, Birmingham, B15 2TL, U.K.*

Recent advances in chemotherapy (CT) have led to the prospect of cure for the majority of men with advanced testicular cancer. We have studied the long-term physical side-effects of such treatments. Thirty-six patients (pts) who had been treated with CT for testicular cancer in the period 1979–1983 and who remained disease-free were asked to take part in the study; 24 agreed to do so. Mean age of study pts was 33 (range 20–51), median time since start of CT was 33 months (6–63). All pts had received *cis*-platinum (CPt), bleomycin (B) and vinblastine (V). Mean doses given (range) were CPt 647 mg (300–1190), B 320 mg (135–675), V 75 mg (40–160). Four pts had also received etoposide and 3 pts actinomycin-D. Median number of courses of CT given was 3.5.

*Renal function*: Prior to CT all pts had normal serum urea and creatinine. After CT 2 had raised urea and 3 raised creatinine. Creatinine after CT showed a strong correlation with total dose of CPt. There was no correlation with age or time since treatment. *Endocrine function*: All pts had normal serum testosterone and free-T4. 11/21 (52%) had raised TSH, 16/24 (67%) had raised LH and 18/24 (75%) had raised FSH. Endocrine dysfunction was more severe in older patients. *Audiometry*: Prior to CT 5 pts had audiometric testing. Four were normal, 1 showed unilateral hearing loss. The majority of pts had objective high-tone hearing loss with 50% having <20 db mean hearing loss for both ears at 6–8 khz. Seven pts (29%) had subjective hearing loss. Extent of high-tone hearing loss was strongly correlated with total dose of CPt but not with age. *Semenalysis*: 4 pts had no semenalysis. All pts not azoospermic on first testing were asked to have repeat semenalysis; 13 agreed. Thus 33 semenalyses were done in 20 pts. Total dose of CPt and V were strongly correlated with reduced sperm counts (SC), reduced motility and reduced % of motile sperm. There were no correlations with age. *Pulmonary Function*: Peak flow rate and CO transfer factor were below predicted for height, age and ethnic origin in more patients than would be expected by chance although only in one pt was CO transfer factor reduced below 2 standard deviations of predicted and there was no correlation with dose of bleomycin. Inspiratory capacity, vital capacity and effective alveolar volume were within the predicted range in all pts but each showed significant inverse correlations with total dose of bleomycin.

CT for testicular cancer causes long-term disturbance of renal, endocrine, pulmonary, audiometric, and reproductive function which is largely dose-related. Except for hearing loss this disturbance is asymptomatic but further follow-up is required to determine whether these abnormalities have any future significance.

### Altered size distribution of Ha-*ras* restriction fragments in patients with colorectal cancer

D. Wynford-Thomas, F.S. Wyllie, N.R. Lemoine, G.T. Williams, E.D. Williams & V. Wynford-Thomas

*Cancer Biology Unit, Department of Pathology, University of Wales College of Medicine, Cardiff, UK.*

The human Ha-*ras* oncogene locus demonstrates a Bam H1 restriction fragment length polymorphism (RFLP) due to variation in copy number of a 28bp 3′ tandem repeat. Studies of patients with a variety of tumours have suggested an association of certain rare alleles with susceptibility to cancer. We have analysed leucocyte DNA from 15 patients with a *single* histological tumour type – adenocarcinoma of the colon/rectum – and from 13 controls with no family history of cancer. Southern blots of Bam H1 digested DNA were hybridised with a 32P labelled 6.6kbp genomic Ha-*ras* probe. Restriction fragment sizes were estimated by reference to a HindIII $\lambda$ digest and to 2 internal standards (95% confidence limits: $\pm$0.15 kbp). Leucocyte DNA in both patients and controls showed the expected RFLP with a predominant (6.6 kbp) and a variety of less frequent (6.1–8.4 kbp) alleles. While there was little difference in the overall *range* of sizes there was a major change in the shape of the distribution. The frequency of the predominant 6.6 kbp allele was reduced from 19 out of 26 (73%) in controls to 9 out of 30 (30%) in cancer patients ($X_2 = 8.7$; $P < 0.01$), with a corresponding 'flattening' of the distribution. (No estimate of 'rare' allele frequencies was attempted.) While our control data agrees with previous reports, our data on colorectal cancer patients contrasts with previous analyses of myelodysplasia and a mixed tumour series, which showed no significant decrease in the frequency of the predominant allele in leucocyte DNA. We suggest that the underrepresentation of the 6.6 kbp allele observed here indicates a specific association between Ha-*ras* RFLP and susceptibility to colorectal cancer and demonstrates the need to evaluate this association for individual tumour types.

### The thyroid follicular epithelial cell as a recipient for DNA transfection

N.R. Lemoine & D. Wynford-Thomas

*Cancer Biology Unit, Department of Pathology, University of Wales College of Medicine, Heath Park, Cardiff CF4 4XN, UK*

With the ultimate aim of developing an immortal epithelial cell line as a test system for detection of epithelial-specific human oncogenes by DNA transfection, we have investigated the uptake and expression of exogenous DNA by human thyroid epithelial (follicular) cells. Primary cultures of normal thyroid follicular cells (free of fibroblasts as judged by electron microscopy) were transfected in suspension or monolayer culture by calcium phosphate co-precipitation with a plasmid containing the SV40 genome together with normal human carrier DNA ($0.5\,\mu g + 20\,\mu g$ respectively per $5 \times 10^5$ cells). The frequency of cells expressing SV40 sequences was assessed 2–7 days after transfection by immunodetection of nuclear large T antigen using antibody PAB419 together with an indirect immuno-operoxidase procedure. In DME/F12 medium containing 10% FCS we observed a frequency of $10^{-4}$ positive cells, which remained unchanged for up to 7 days. Addition of thyroid stimulating hormone (TSH) $10\,mU\,ml^{-1}$, 20 min before transfection increased this tenfold to $10^{-3}$. Inclusion of a lysosomal inhibitor ammonium chloride (20 mM) resulted in a further improvement to $2 \times 10^{-3}$. These results support our hypothesis that the unusual phagocytic property of the thyroid follicular cell when stimulated by TSH may make this an especially suitable epithelial cell for transfection. We are currently exploring the frequency of stable expression using this system.

### Optimisation of the NIH3T3 focus assay for detection of activated oncogenes

N.R. Lemoine, V. Wynford-Thomas & D. Wynford-Thomas

*Cancer Biology Unit, Department of Pathology, University of Wales College of Medicine, Cardiff, UK.*

Although DNA mediated induction of transformed foci in NIH3T3 monolayers has been widely used as an assay for activated oncogenes in human tumours, its usefulness has been limited by a high and variable incidence of background spontaneous foci. We set out to correct this by examining the influence of (a) serum concentration, (b) feeding interval, (c) medium composition and (d) plastic surface, on (i) the incidence of spontaneous foci, and (ii) the incidence of true foci induced by transforming sequences, using plasmid $pSV_2neoEJ$ containing activated Ha-*ras* as a positive control. Using 'high background' NIH3T3 cells (which had been serially-passaged for 6 months) in standard culture conditions – 5% calf serum (CS), with a 3 day feeding interval using DME medium on 90 mm Nunc dishes – we observed a spontaneous focus incidence of 20/dish and a true focus incidence of 21/dish. (a) Simply adjusting the serum concentration was not successful since concentrations below 5% failed to support a viable monolayer while higher concentrations increased the spontaneous focus rate. (b) Keeping the serum concentration at 5% but reducing the feeding interval to 1 day, however, dramatically reduced the spontaneous focus incidence (to 0/10 dishes) giving a reproducibly uniform monolayer. Furthermore the true rate was improved to 55/dish. (c), (d) Use of a 'richer' medium (DMEM: F12/1:1) and Falcon dishes both gave further improvements in the true focus rate (up to 154/dish) with no deterioration in the background.

Our results demonstrate a dramatic improvement in the 'signal to noise' ratio of the focus assay by simple adjustment of culture conditions without recourse to recloning.

**Heterogeneity of c-*myc* expression in human tumour cells detected by *in situ* hybridisation**

J. Boultwood, E.D. Williams & D. Wynford-Thomas

*Cancer Biology Unit, Department of Pathology, University of Wales College of Medicine, Cardiff, UK.*

*In situ* hybridisation represents a useful complementary technique to Northern blotting for analysis of oncogene expression in human tumours since it offers the potential to detect aberrant expression in minor subpopulations of cells. Comparison of the various techniques available using the promyelocytic leukaemia cell line HL60 (which is known to overexpress c-*myc*) demonstrated the superiority of single stranded c-RNA probes generated by SP6 polymerase. Using $^{32}$P labelled c-*myc* cRNA and emulsion autoradiography we observed an unexpected heterogeneity in expression of this oncogene in asynchronous, undifferentiated HL60 cultures. While the majority showed a range of intensities (from 10–100 grains/cell after 7 days exposure), 20% of cells were intensely labelled (>200 grains/cell) whereas 20% were indistinguishable from background (<3 grains/cell). In a preliminary study of medullary carcinoma of the thyroid, using both frozen and paraffin sections. c-*myc* was not detectable above the background, non specific signal (<3 grains/cell) in 4 out of 5 tumours, and in normal thyroid and other control tissues. One case, however, was clearly positive. In this tumour, while a weak signal (10–20 grains/cell) was observed over the majority of the cells, one nodule (representing ~10% of the total tumour area observed in the section) was intensely positive (>200 grains/cell). These examples of heterogeneity of oncogene expression clearly demonstrate the additional information which can be obtained by *in situ* compared with homogenate-based hybridisation.

**Measurement of 5-methylcytosine in DNA**

J. Catania[1], B.C. Keenan[2], G.P. Margison & D.S. Fairweather[1]

[1]*Department of Geriatric Medicine, Withington Hospital and* [2]*Department of Carcinogenesis, Paterson Institute for Cancer Research, Manchester M20 9BX, UK.*

5-methylcytosine (5-MeC) in DNA may be involved in the control of eukaryotic gene expression (A. Razin & A.D. Riggs, *Science*, **210,** 604 (1980)). Quantitation of this product involving the degradation of DNA to purine and pyrimidine bases has been inaccurate because deamination of cytosine and 5-methylcytosine occurs during the hydrolysis procedure (J.P. Ford *et al.*, *J. Biol. Chem.*, **255,** 7544 (1980)). We have therefore undertaken a systematic study of the hydrolysis of DNA by hydrofluoric acid (HF). Separation of the bases was achieved using a Nucleosil 5 SB column eluted with 10 mM sodium acetate pH 4.0 containing 30% methanol, and quantitation at 280 nm was by automated peak area integration. Deoxycytidine and 5-methyldeoxycytosine were shown not to undergo detectable levels of deamination during prolonged periods (up to 24 h) at 80°C in 48% HF. Kinetic studies showed that the release of purine and pyrimidine bases was complete by 4 h under these conditions. Analysis of the 5-MeC content of DNA from Herring testis and Calf thymus gave 5-MeC levels of 9.62% and 6.67% respectively i.e. very close to the literature values of 10.8% and 6.6% (G.D. Fasma (ed.), *Handbook of Biochemistry and Molecular Biology*, 3rd Edn. p. 241 (1976)). This method is ideally suited for the determination of the overall cytosine methylation levels in DNA. (HF is hazardous)

**The *ras* oncogene and metastasis**

J.S. Wallace, A.J. Hayle, A.J. Syms, M. Evans, D. Tarin & K.A. Fleming

*Nuffield Department of Pathology, University of Oxford, John Radcliffe Hospital, Oxford OX3 9DU, UK.*

Transfection of cells with cloned genes or total genomic DNA offers opportunities for studying the regulatory disturbances responsible for aspects of neoplastic behaviour. We have used this method to examine whether incorporation of the cloned 6.6 kb fragment of the mutated c-Ha-*ras* human oncogene into the genome of 3T3 fibroblasts can confer metastatic capability on these cells. 3T3 cells co-transfected with the mutated *ras* gene and the neomycin resistance plasmid psv2-neo were selected by culture in neomycin. On subcutaneous inoculation into MF1 nude mice these cells proved to be tumorigenic with short latent periods (~14 days). However, there was no evidence of spontaneous metastasis at autopsy, or on histological examination of the lungs and other organs, 90 days after inoculation. Intravenous inoculation of cells transfected with *ras* showed that they were clonogenic in the lungs but cells transfected with psv2-neo alone were not. Successful incorporation of the oncogene into the clones obtained by neomycin selection was confirmed by Southern blotting and expression was demonstrated by immunoprecipitation of the *ras*$^{H}$ protein.

The results in this experimental system indicate that transfection of a mutated *ras* oncogene into non-neoplastic 3T3 cells is not by itself sufficient to initiate spontaneous metastatic behaviour but can make the cells tumorigenic and capable of colonising the lungs if they are inoculated intravascularly.

**Phenotypic heterogeneity of B-cell antigen expression and HLA class II antigens in B-cell-non-Hodgkin's lymphoma (B-NHL)**

D.B. Jones, K. Moore[1] & D.H. Wright

*University Department of Pathology and* [1]*Regional Immunology Service, General Hospital, Southampton SO9 4XY, UK.*

We describe the results of an investigation in frozen section of 50 cases of B-NHL with a panel of 15 monoclonal antibodies directed to B cell surface antigens and to HLA sublocus products. As a group, B cell tumours displayed a considerable variation in staining intensity with different B cell surface antigens and this was also apparent between tumours of the same histological type. Heterogeneity was also apparent when cases of B-NHL were examined with antibodies identifying different epitopes of a single surface molecule. This type of heterogeneity was most marked with antibodies directed towards the CD22 antigen widely expressed on normal B cells. Functional expression of different CD22 epitopes, therefore, varies in neoplastic cells. Antibodies specific for the HLA Class II sublocus products, DP, DR and DQ, all generally expressed on normal B cells, similarly showed heterogeneity in B-NHL: commonly, the loss of a single sublocus product from the tumour cell surface or, rarely, the expression of one sublocus product only. These data indicate that in B-NHL the tumour population does not always mimic exactly, in phenotype, the equivalent normal B cell population and has implications in the construction of monoclonal panels for the routine diagnosis of B cell tumours.

**Analysis of sugar specific cellular glycoproteins from Hodgkin lymphoma and other lymphoma/leukaemia cell lines**

D.J. Flavell, D.B. Jones & D.H. Wright

*Department of Pathology, Southampton General Hospital, Southampton SO9 4XY, UK.*

Studies aimed at determining the histogenesis of the Reed–Sternberg cell, the putative neoplastic cell of Hodgkin's lymphoma, have been hampered in the past by difficulties encountered in preparing pure suspensions of such cells. Recently however, a number of cell lines derived from Hodgkin involved tissue have become available and whilst it is not certain that these genuinely represent the Reed–Sternberg cell population, they do share certain characteristics in common with their *in vivo* counterparts. We have undertaken to analyse by SDS-polyacrylamide gel electrophoresis (PAGE), Western blotting and lectin probing, the cellular glycoprotein profiles of four Hodgkin cell lines (L428 and L591, Diehl *et al.*, *Cancer Treatment Rep.*, **66**, 615, 1982; Ho and Co; Jones *et al.*, *Haematol. Oncol.*, **3**, 133, 1985) and compare these with profiles obtained for cell lines of established lymphoid, myeloid or monocytoid origin. Six $^{125}I$ labelled lectins representing the most common carbohydrate specificities were used to probe nitrocellulose membranes of SDS-PAGE separated detergent solubilised cellular glycoproteins for each cell line studied. Lectins binding to specific bands were visualised by autoradiography and molecular weights ($M_r$) calculated by reference to a standard calibration curve. The complex glycoprotein profiles obtained for the Hodgkin cell lines studied suggest these cells to be of lymphoid origin, an observation in agreement with both immunophenotypic and immunoglobulin/T cell receptor rearrangement studies. Work is now in progress to raise monoclonal antibodies against specific glycoprotein bands of interest for use as immunocytochemical probes on tissue section.

**Monoclonal anti-light chain idiotype as a tumour-specific probe for human B cell lymphoma**

M. Wrightham, A.L. Tutt, M.J. Glennie, T.J. Hamblin, G.T. Stevenson & F.K. Stevenson

*Lymphoma Research Unit, Tenovus Laboratory, General Hospital, Southampton SO9 4XY, UK.*

Preparation of monoclonoclonal anti-idiotype reagents for monitoring and therapy of B cell neoplasms is dependent on a supply of idiotypic Ig as immunogen. This is not readily available from tumours which do not secrete or display high levels of Ig. Such patients frequently have low levels of urinary monoclonal light chain of neoplastic origin. The object of this study was to investigate whether monoclonal antibody raised against isolated light chain idiotype also recognised determinants in intact (heavy + light chain) idiotypic Ig, allowing urinary light chain to serve as alternative immunogen. A hybridoma was selected secreting antibody which recognised idiotypic $\lambda$ of patient L.P., but not normal $\lambda$ chains by a preliminary screen, and which also reacted with idiotypic IgM$\lambda$ on the patient's tumour cells. The antibody did not recognise normal tonsil cells (immunoperoxidase staining) or a panel of IgM$\lambda$ paraproteins (direct binding ELISA). The antibody was characterised more fully using an ELISA system based on inhibition of antibody binding to idiotypic light chain coated onto a solid phase by various $\lambda$-containing species in the free solution phase. By this criterion the antibody recognised 1 in $2 \times 10^4$ molecules of pooled normal $\lambda$ chains, confirming its specificity for a private idiotope. The target epitope appeared to be less available in dimeric light chain than in the monomer or idiotypic IgM. This epitope is clearly distinct from that recognised by another monoclonal antibody specific for combined (heavy + light chain) idiotype, as the two showed additive binding to IgM$\lambda$ L.P. in a direct binding ELISA system. Such anti-light chain idiotype reagents should be applicable to monitoring and possibly therapy of B cell neoplasms.

**Emergence of immunoglobulin variants following treatment of a B-cell leukaemia with anti-idiotype: saporin immunotoxin**

M.J. Glennie[1], H. McBride[1], A.T. Worth[1], F. Stirpe[2], P.E. Thorpe[2] & G.T. Stevenson[1]

[1]*Lymphoma Research Group, Tenovus Laboratory, Southampton SO9 4XY and* [2]*ICRF, Lincoln's Inn Fields, London WC2A 3PX, UK.*

An immunotoxin (IT) composed of monoclonal anti-idiotype (Id) coupled via disulfide linkage to the plant ribosome-inactivating protein saporin, has been investigated in the treatment of guinea pig $L_2C$ leukaemia. *In vitro* this conjugate remained toxic to $L_2C$ cells, as measured by the incorporation of [$^3$H] leucine into protein, at below $10^{-10}$ M and was respectively >10,000 and 900 times more potent than free saporin and saporin coupled to an antibody of irrelevant specificity. In therapy a single s.c. injection of reactive IT given 24 h after an i.p. inoculum of $10^5$ $L_2C$, increased the mean survival of animals from 15 days to 30 days. Under the same conditions control reagents, including free saporin, anti-Id alone or saporin coupled to a non-reactive antibody, did not prolong animal survival. All $L_2C$ cells emerging after IT therapy showed altered immunoglobulin (Ig) expression which rendered them non-reactive with the IT. Predominantly $L_2C$ cells had lost $\mu$ heavy chain production leaving them negative for intracellular, surface and secreted IgM, but still positive for idiotypic light chain production. In addition a minor group of $L_2C$ variants did express normal levels of IgM but with an altered or mutated Id which rendered them non-reactive with the IT.

Our previous immunotherapy investigations using monoclonal anti-Id alone have not revealed these Ig variants. We suggest that it is the increased selective force exerted by the highly potent IT which allowed non-reactive populations to emerge. Such cells, particularly those of an Ig negative phenotype, could prove a major obstacle to the application of IT therapy in humans.

**Anti idiotypic mechanisms lead to tumour dormancy and protection in a murine lymphoma, $BCL_1$**

A.J.T. George & F.K. Stevenson

*Lymphoma Research Unit, Tenovus Research Laboratories, Southampton General Hospital, Southampton, SO9 4XY, UK.*

Idiotypic determinants on the surface immunoglobulin of B cell tumours are tumour-associated antigens and therefore present a target for anti-idiotypic attack. Immunization of mice with idiotypic IgM from the syngeneic $BCL_1$ lymphoma has been used to generate anti-idiotypic responses and to follow the effect on tumour development. Such immunization protects specifically against challenge with some mice surviving >6 months. Spleens from long-term survivors with no macroscopically visible tumour, when examined with anti-idiotypic antibody, showed a range of apparently dormant tumour occupying 2–50% of the spleen. On the passage of these spleen cells into naive mice, $BCL_1$ tumour developed and killed the recipients in times indistinguishable from routine tumour passage. Analysis of possible mechanisms of suppression in immunized mice identified low levels of cytotoxic anti-idiotypic antibody in serum and splenic T cells which proliferated specifically in response to idiotypic IgM. Only low levels of cytotoxic T cells were found. Passive transfer studies demonstrated a major role for antibody in protection against tumour. Tumour arising in immunized animals shows a variable pattern of expression of idiotypic IgM at the cell surface, although it was always present in the cytoplasm. Passage of a low number of cells from one such emergent tumour allowed the isolation of a stable variant tumour, SNAG 1, which lacked surface idiotype and IgM. In spite of containing cytoplasmic idiotype this variant failed to secrete idiotypic IgM *in vivo* or *in vitro*. This study raises the possibility of attempting a similar immunization in human patients with low grade lymphoma who might be capable of mounting an anti-idiotypic response.

### Idiotypic immunoglobulin as an indicator of residual disease in multiple myeloma following treatment with high dose melphalan

J. Thompson[1], P. Selby[2], V. Anderson[3], T.J. McElwain[2] & G.T. Stevenson[3]

[1]*Wessex Medical Oncology Unit, Southampton General Hospital, Southampton;* [2]*Royal Marsden Hospital, Sutton and* [3]*Tenovus Research Laboratory, Southampton, UK.*

Patients in apparent complete remission† (CR) from multiple myeloma following treatment with high dose melphalan were monitored for residual disease using a sensitive anti-idiotype (Id) enzyme-linked immunosorbent assay (ELISA) capable of detecting low levels of tumour-derived idiotypic Ig. High titre anti-Id anti-serum was prepared for each patient in rabbits, using idiotypic myeloma Ig isolated from pre-treatment sera as a source of immunogen. Reactivity towards common immunoglobulin (Ig) determinants was removed by extensive absorption against pooled human IgG and/or IgA as appropriate, and then antibodies were affinity purified by elution from a column of the patients' purified idiotypic Ig. In the ELISA these purified anti-Id antibodies were used as a coating layer to capture the patients idiotypic Ig from test sera, before detecting with an enzyme-labelled antihuman Ig antibody. The specificity of each anti-Id reagent was fully established against pooled human serum and irrelevant immunoglobulins.

Ten patients are under investigation in the current study. All achieved CR as judged by conventional criteria, including the absence of a monoclonal band from electrophoretic strips of the patients' sera. Despite these indications the sensitive ELISA system presented has shown that in all 6 patients monitored so far, idiotypic myeloma protein remained in the circulation throughout the post-treatment period at levels of $<1,000\,\mu g\,ml^{-1}$. Three of the 6 patients have since relapsed clinically. We suggest that the monitoring system described could be useful in evaluating CR in the research context. (†No detectable paraprotein in serum, no urinary monoclonal light chain excretion and bone marrow morphologically normal.

### Intensive chemotherapy (MACOBLE) for aggressive non-Hodgkin's disease

G.J. Forrest[1], D. Cunningham[1], T. Young[1], R. Moss[1], I.L. Evans[2] & M. Soukop[1]

[1]*Department of Medical Oncology, Royal Infirmary, Glasgow and* [2]*Department of Haematology, Western Infirmary, Glasgow, UK.*

Aggressive non-Hodgkin's lymphomas have a poor prognosis unless treated by intensive chemotherapy. Between 1983 and 1986, 53 patients (49 previously untreated) received intensive chemotherapy with cyclophosphamide, $750\,mg\,m^{-2}$ i.v.; adriamycin, $40\,mg^{-2}$ i.v.; bleomycin, 15 mg i.m. on day 1, etoposide $100\,mg\,m^{-2}$ i.v. on days 1 and 2; vincristine, 2 mg i.v.; bleomycin 15 mg i.m.; methotrexate, $300\,mg\,m^{-2}$ i.v. bolus and methotrexate, $1.2\,g\,m^{-2}$ 12-hourly infusion with folinic acid rescue on day 10. Oral prednisolone, 100 mg was also given days 1–5 q 3-weekly (MACOBLE).

Twenty-nine patients had Stage IV disease, 8 Stage III, 11 Stage II and 5 Stage I disease. Immunocytochemical markers were performed in 36 patients (23 T cell, 13 B cell).

Twenty-two (41%) patients achieved CR with a total response rate to chemotherapy of 75%. Median survival of patients reaching CR has not been achieved but will be in excess of 16 months. The median survival of patients achieving PR or stable disease/PD was 11 months and 3 months respectively. Four patients with major PR successfully achieved CR after radiotherapy to areas of residual bulk disease. The major toxicity was myelo-suppression-median WCC $1.2\times10^3\,mm^{-3}$ and platelet $116{,}000\,mm^{-3}$. The major prognostic indicator was achieval of CR. T cell markers were not an adverse prognostic indicator. The MACOBLE regimen is a useful therapy in aggressive non-Hodgkin's lymphomas.

### Treatment of refractory and relapsed non-Hodgkin's lymphoma (NHL) with ifosfamide (I), methotrexate (M) and etoposide (VP16)

G. Sangster & M.J. Leyland

*Department of Clinical Haematology, East Birmingham Hospital, Birmingham B9 5ST, UK.*

Fourteen patients (12 male, 2 female), mean age 50 years (range 25–68), with refractory or relapsed NHL (12 patients), chronic lymphocytic leukaemia (CLL, 1 patient) or Hodgkin's disease (HD, 1 patient) were treated with ifosfamide (I) $1\,g\,m^{-2}\,day^{-1}$ for 5 days by 2 h infusion (5 patients) or $4\,g\,m^{-2}$ by 24 h infusion for 1 day (9 patients), etoposide (VP16) $100\,mg\,m^{-2}\,day^{-1}$ for 3 days by 2 h infusion and methotrexate (M) $30\,mg\,m^{-2}$ bolus on days 3 and 10. Courses of IMVP-16 were repeated every 21–38 days. Patients receiving ifosfamide 24 h infusion were given mesna (total dose $7.2\,g\,m^{-2}$) as a 36 h infusion in addition to a high fluid intake to prevent ifosfamide-induced urotoxicity. Mesna (total daily dose $1.2\,g\,m^{-2}$) was given by bolus injections to those patients receiving fractionated ifosfamide. Six NHL patients had high-grade (HG), and 6 had low-grade (LG) histology (Kiel classification). Ten/twelve NHL patients had stage III/IV disease and 5 had bone marrow infiltration. Prior therapy included CHOP/MTX in 11 patients. Four patients with LG and 1 with HG-NHL had been in complete remission (CR, duration range 2 months–4 yrs) after CHOP; 7 NHL patients had progressed on CHOP after an initial response. Two patients died of progressive NHL and/or sepsis during the first course of IMVP-16. Twelve patients received at least 2 courses and were evaluable for response using standard WHO criteria. Four patients (3 NHL, 1HD) showed progressive disease (PD) after 2 courses and 7 patients with less than a partial response (PR) failed to achieve PR or CR during 2 further treatment cycles. One patient with low-grade NHL relapse in small bowel had only minimal residual disease (local mesenteric nodes) after surgery and has remained in clinical CR for $\geqq 15$ months. A nadir neutrophil count $\leqq 500$ or platelet count $\leqq 50{,}000$ was seen in 23/40 and 12/40 courses respectively. Five episodes of infection associated with neutropenia were seen. Nausea and vomiting was generally mild and there was no urotoxicity. The results of this study suggest that salvage therapy with IMVP-16 in HG-NHL may have very limited value in patients who have not had a durable response to first-line therapy.

### Management of localised (stage I+II) non-Hodgkin's lymphoma (NHL)

R.E. Taylor, S.G. Allan, G.L. Ritchie, G.R. Kerr, J.F. Smyth & R.C.F. Leonard

*Department of Clinical Oncology, Western General Hospital, Edinburgh, UK.*

Between Jan. 1974 and Dec. 1983, 177 patients (113 high grade and 64 low grade) stage I+II NHL were treated with surgical excision alone (4 patients) or excision/biopsy combined with irradiation (125 patients), chemotherapy (24 patients) or both (24 patients). Seventy-five (42.4%) presented with nodal and 102 (57.6%) with extra-nodal disease. Actuarial survival for stage I patients was 75.2% at 5 years and 71.7% at 10 years and for stage II patients 64.3% at 5 years and 58.9% at 10 years. For patients with stage II and bulky ($\geq$5 cm) stage I, and gastrointestinal, high grade NHL, there was a non-significant trend towards prolongation of survival+RFS for patients treated with chemotherapy alone or together with irradiation, compared with irradiation alone. For patients responding completely to chemotherapy, irradiation of bulky sites did not appear necessary. For irradiated patients, local control was achieved in 51/57 (89%) with low grade and 79/91 (87%) with high grade NHL, and for bulky high grade NHL in 31/40 (77.5%) treated with $<$40 Gy and 6/6 (100%) with $\geq$40 Gy. There was no advantage for extended compared with involved fields. Survival was significantly prolonged for low grade compared with high grade patients, for patients aged $<$60 and for those with a complete response to primary therapy. For extranodal NHL, actuarial survival at 5 years was 80% for thyroid, 87.5% for low grade and 42.5% for high grade gastrointestinal, and 82.4% for stage I and 0% for stage II Waldeyer's ring.

### A study of the gastro-intestinal tract in patients with B cell neoplasms

G. Smith[1], I. Chesner[3], J. Crocker[2], M.J. Leyland[1] & P. Asquith[3]

[1]*Department of Haematology;* [2]*Department of Histopathology and* [3]*The Metabolic Unit, East Birmingham Hospital, Bordesley Green East, Birmingham, UK.*

A review of data collected at this hospital from patients with chronic lymphocytic leukaemia (CLL) and non-Hodgkin's lymphoma (NHL) suggested that iron deficiency was a common finding. In order to evaluate the cause, fifteen patients with CLL and NHL, not primarily of gastro-intestinal origin, were extensively investigated in the Metabolic Unit. Standard tests of absorptive function were performed together with endoscopy, sigmoidoscopy and aspiration of duodenal contents. Duodenal juice was sent for bacterial culture and estimation of immunoglobulin levels. A lactulose hydrogen breath test was also performed. Gastric, duodenal and rectal biopsies were stained using immuno-gold-silver staining (IGSS) technique. This immunostaining method has much enhanced sensitivity for demonstrating antigens in paraffin sections as compared with peroxidase-anti-peroxidase methods, and reliably stains surface as well as cytoplasmic immunoglobulin. Using this technique, seven patients were clearly demonstrated to have infiltration of the gastrointestinal mucosa by monoclonal B cells at one or more sites. Six patients were found to be iron deficient with impaired iron absorption. Five of these had gastrointestinal involvement. The hydrogen breath test indicated bacterial contamination of the small intestine in nine patients. This has been confirmed by positive culture of duodenal contents in four cases. There was associated hypogamma-globulinaemia in four cases. We conclude that occult involvement of the gastrointestinal tract is a common finding in patients with B cell neoplasms and is associated with impaired absorptive function and possibly impaired mucosal immunity.

### Non-Hodgkin's primary lymphoma of the gastrointestinal tract

R.R. Moss[1], S. Allan[3], T. Young[1], R. Taylor[3], D. Cunningham[1], G. Forrest[1], R.F. Leonard[3], M. Soukop[1] & S.B. Kaye[2]

[1]*Department of Oncology, Glasgow Royal Infirmary;*
[2]*Department of Oncology, Western Infirmary, Glasgow and*
[3]*Department of Oncology, Western Infirmary, Edinburgh, UK.*

A study was made of 81 patients with primary gastro-intestinal lymphoma. Forty-four per cent of tumour occurred in the stomach or duodenum; 44% in the small bowel or mesentery; 5% in the colon or rectum; 2% in the ileocaecal region. In the remainder it was not possible to determine the site of origin. The Rappaport histological classification was used. The commonest histological subtype was diffuse histiocytic (48%). This group had a poor prognosis with a median survival of 14 months; in contrast, the lymphocytic lymphomas fared better, irrespective of nodularity or differentiation. The patients were staged according to the system proposed by Blackledge. Stage I had the best prognosis (5-year survival 73%); in contrast, there were no stage 4 5-year survivors. Eighty-four per cent of patients had some form of surgical resection; prognosis was better in those where the resection was complete. The majority of patients received in addition radiotherapy or chemotherapy; survival in the surgery/radiotherapy group was better than in the surgery/chemotherapy group. Overall 5-year survival was 43%.

### Increasing incidence of oesophageal carcinoma; in which sites and which histological types?

J. Powell, J.E. Robertson & C.C. McConkey

*Clinical Cancer Monographs, Regional Cancer Registry, Q.E. Medical Centre, Birmingham B15 2TH, UK.*

Oesophageal carcinoma is such a rare tumour (5 per 100,000) that population based studies on changes in the incidence by both sub-site and histology have not hitherto been possible. In a study of 6,398 cases occurring in the West Midlands Region over the 25 year period 1957–1981 the numbers were sufficient to enable marked differences to be observed within classifications by both parameters.

The categorisation by site was into upper, middle and lower thirds. Tumours of doubtful origin were reviewed and those arising in either the hypopharynx or cardia were rigorously excluded. It was not possible to specify a subsite within oesophagus for ~12% of the cases, but as this proportion has remained relatively constant throughout the period it is unlikely to have introduced any bias. The histological breakdown was into squamous, adenocarcinoma, anaplastic and 'no histological confirmation' (30%). To compensate for changes in the age structure of the population, age-standardised incidence rates were used.

Results indicate a steady increase in incidence of the middle and lower thirds for both sexes. Tumours of the upper third and of unspecified site remained relatively stable, indicating that this increase is a real one and not the result of changes in classification. The increase in the middle third occurred in squamous and in adenocarcinomas and in both sexes. In anaplastic carcinomas the increase was only in females. In the lower third there was an increase in squamous cell carcinoma in women but not in men. For adenocarcinomas the rates increased in both sexes and was highly significant in men. Anaplastic carcinomas showed little change.

**Inflammatory or malignant infiltration of rectal tumours – Is there an alternative to digital assessment?**

J. Beynon, D.M.A. Foy, J.L. Channer, J. Virjee & N.J.McC. Mortensen

*Departments of Surgery, Medical Physics, Pathology and Radiology, Bristol Royal Infirmary, Bristol BS2 8HW, UK.*

Malignant fixation in rectal tumours is associated with a worse prognosis and particularly a higher incidence of local recurrence. Distinguishing between these two types of fixation by digital examination may be difficult. Using endoluminal ultrasonograph) (ELU) we have compared pre-operative ultrasonic measurements of the depth of the invasion with histopathological estimates of depth measured from both prepared sections and operative specimens. Ultrasonic examinations were performed with a rotating endoprobe using 5.5 MHz and 7.0 MHz transducers, selecting images at the site of maximum depth of tumour. In 35 patients a comparison of ELU tumour depth was made with maximum depth measured from the histological section. The coefficient of correlation was 0.63 ($P<0.001$). In 27 patients it was possible to compare ELU with tumour depth measured from the resected specimen, coefficient of correlation – 0.80 ($P<0.001$). Pre-operative ELU assessments of the depth of invasion of rectal tumours are accurate when compared with histology. ELU may thus provide an objective method for differentating between inflammatory and malignant infiltration.

**Endosonography for the assessment of para-rectal lymph node involvement in rectal cancer**

J. Beynon, D.M.A. Foy, J.L. Channer, N.J.McC. Mortensen & J. Virjee

*Departments of Surgery, Medical Physics, Pathology and Radiology, Bristol Royal Infirmary, Bristol, BS2 8HW, UK.*

Assessment of para-rectal lymph node involvement in rectal cancer continues to be a problem. Previous studies have shown digital examination correctly identifies only 50% of involved lymph nodes while computer tomography is also disappointing in this field. Endoluminal rectal ultrasound has been used to assess the involvement of pararectal lymph nodes in 66 patients with primary rectal cancer. Examinations have been performed with a rotating endoprobe and 5.5 MHz and 7.0 MHz transducers. Subsequently 57 resections were performed and histological assessment compared with ultrasonic data. Sonographically, involvement was correctly predicted in 22 cases with 7 false positives, while 24 cases were negative with 4 false negatives. The coefficient of correlation between ultrasonic examination and histopathology was 0.62 ($P<0.001$). The accuracy for predicting lymph node metastases was 81%, the sensitivity 85%, specificity 77% and the predictive value 76%. Endoluminal rectal ultrasound is an accurate method of assessing pararectal lymph node involvement in rectal cancer and its use pre-operatively enables a more accurate staging to be performed.

**Properties of a breast carcinoma associated antigen defined by the monoclonal antibody NCRC-11**

M.R. Price, G. Crocker, S. Edwards & R.W. Baldwin

*Cancer Research Campaign Laboratories, University of Nottingham, Nottingham, NG7 2RD, UK.*

NCRC-11 is an anti-human breast carcinoma monoclonal antibody which defines a high mol. wt glycoprotein antigen (>400 kD) and the expression of this antigen in breast carcinomas correlates with patient survival (Ellis *et al.*, *Br. Med. J.*, **290**: 881, 1985). The antigen has been purified from detergent extracts of breast carcinomas by immunoadsorbent chromatography, and shown to be a wheat germ agglutinin-binding glycoprotein, which is susceptible to proteolysis with pepsin or papain although NCRC-11 antibody binding is unaffected by heat (100°C for 5 min) or neuraminidase treatment. In addition to its presence in tumours, the NCRC-11 antigen is also a product of specialized normal epithelia (being particularly associated with luminal surfaces of secretory epithelia), although in healthy individuals it is not found in the circulation. However, in breast cancer patients, the breast tissue architecture is disrupted sufficiently by the developing tumour to allow products from the tumour to have access to the circulation. Thus, NCRC-11 antigens have been identified in breast cancer patients' serum both by immunoblotting techniques and by sandwich radio-immunoassay. The findings indicate that the NCRC-11 antigen has potential as a diagnostic and prognostic marker in breast cancer.

**Expression of histocompatibility antigens and characterisation of mononuclear cell infiltrates in human renal cell carcinomas**

M.O. Symes, D. Heinemann & P.J.B. Smith

*Departments of Surgery and Urology, University of Bristol, UK.*

Neoplastic tissue was obtained at operation from 10 renal cell carcinomas, from the adjacent normal kidney in 6 cases and from 1 other normal kidney. The biopsies were snap frozen in liquid nitrogen and sections were subsequently stained with monoclonal antibodies against major histo-compatibility complex antigens, Class I and II, and several types of mononuclear cell, by the indirect immunoperoxidase method. The degree of staining was graded from heavy 4, through moderate 3, few 2, occasional 1, to nil 0. MHC Ag were consistently expressed, grad 3–4, by the glomerular basement membranes and proximal convoluted tubules of normal kidney, but were absent in 8 of 10 carcinomas. There was a grade 3–4 mononuclear cell infiltration in the stroma of normal kidney and between the carcinoma cells which was composed principally of macrophages. However, in the two carcinomas expressing MHC Ag there was a grade 2–3 infiltration with T lymphocytes. The absence of MHC Ag on carcinoma cells mitigates against attempts to potentiate the patient's immune response to his tumour, e.g. by renal artery embolisation.

**Effector cell populations in antibody-dependent cellular cytotoxicity (ADCC)**

R.J. Dearman, M.J. Glennie & G.T. Stevenson

*Lymphoma Research Group, Tenovus Laboratories, Tremona Road, Southampton, SO9 4XY, UK.*

We have investigated the principal effector cells in human peripheral blood capable of lysing neoplastic B cells (guinea pig $L_2C$) coated with various anti-idiotype (Id) antibodies (Abs). In addition to conventional monoclonal and polyclonal Abs we have employed a range of chimeric univalent Abs consisting of Fab′γ from monoclonal anti-Id covalently coupled to Fc fragments derived from human, rabbit or guinea pig IgG. These derivatives have the advantage of being univalent and therefore non-modulating, and of having interchangeable Fc zones on the one Ab Fab′γ.

Our results suggest that human peripheral blood mononuclear cells contain two populations capable of mediating ADCC. First, a population of non-adherent lymphocytes which were cytotoxic with Abs and Ab derivatives bearing human, rabbit or guinea pig Fc regions, but not with the mouse monoclonal Abs. The performance of these cells was considerably enhanced following overnight pre-incubation in human recombinant interferon γ (γIFN), but was abolished by addition of a monoclonal Ab (3G8) which binds and blocks the Fcγ receptors on human lymphocytes. The second effector population probably contained largely monocytes and was only cytotoxic with mouse monoclonal Abs. Monocyte depletion of effectors by adherence to plastic considerably reduced the cytotoxicity of all monoclonal Abs. Furthermore, and again in contrast to the Abs bearing human, rabbit or guinea pig Fc regions, ADCC by monoclonal Abs was not enhanced by γIFN treatment and was not blocked by the Ab 3G8. Together these results point to two discrete ADCC effector populations: A lymphocyte population which can lyse target cells coated with human, rabbit and guinea pig Abs; and a monocyte population which can recognise mouse Abs.

### Role of MHC class I antigens and the CD3 complex in the lysis of autologous human tumours by T cell clones

T.E. Roberts, U. Shipton & M. Moore

*Department of Immunology, Paterson Institute for Cancer Research, Christie Hospital and Holt Radium Institute, Manchester M20 9BX, UK.*

Peripheral blood lymphocytes (PBL) of 4 patients with malignant effusions were stimulated for 6 days with purified autologous tumour cells, before isolation of the lymphoblasts and cloning by limiting dilution in interleukin-2 (IL2). Forty-five clones were analyzed for cytotoxicity (CTX) against autologous, allogeneic and cell-line targets of known status with respect to expression of major histocompatibility complex (MHC) antigens, estimated by reaction with the W6/32 (anti HLA, −A, −B, −C monomorphic) and TDR31.1 (anti HLA-DR) monoclonal antibodies (McAb). Twenty-one of the 45 clones were cytotoxic, 7 for the autologous target only, the remainder exhibiting various degrees of activity against allogeneic and cell-line targets as well. All clones were $CD3^+$. Autologous CTX was almost always inhibited with W6/32 and OKT3 and allogeneic CTX was also inhibited by these McAbs in some, but not all, effector:target combinations. By contrast anti-K562 activity was never inhibited. The data suggest that to accomplish lysis of autologous and allogeneic targets certain clones require MHC recognition and a functional CD3 complex, while for others with similar target cell repertoires, there is no such requirement. Differential inhibition by W6/32 and OKT3 against autologous and allogeneic targets and K562 indicates that separate receptors are involved in the recognition of fresh tumour and cell-line targets.

It is possible that T cell clones responding to a tumour-associated antigen (TAA) in the context of self MHC antigens can also respond to an allogeneic class I product in the absence of TAA; and/or that aberrant class I antigen expression on autologous tumours accounts for the alloreactivity.

### Abnormally fucosylated haptoglobin in cancer sera

S. Thompson & G.A. Turner

*Department of Clinical Biochemistry, The Medical School, Framlington Place, Newcastle upon Tyne, NE2 4HH, UK.*

Serum protein-bound fucose is frequently elevated in cancer patients (Turner *et al., J. Clin. Pathol.*, **38**, 588, 1985). Possible explanations include the increased production of pre-existing serum glycoproteins and/or alterations in their sugar moieties. The acute phase proteins are known to be elevated in cancer but it is not clear if their gylcosylation is at all altered. We have developed a new method for the removal of fucoproteins from normal and cancer sera using fucose-binding lotus-lectin coupled to agarose. Isolated fucoproteins are eluted from the lectin-agarose using 0.5 M fucose or 5% sodium dodecyl sulphate. They are then analysed by electrophoresis and silver staining. Of several consistent changes in the cancer sera the most striking is a large increase in a component of approximate molecular weight 41,000 kd, especially in patients with more advanced cancers. Two dimensional electrophoresis shows that this component is not α1-acid-glycoprotein and that it appears to be most similar to haptoglobin although slightly more basic. The identity of this molecule as fucosylated haptoglobulin is confirmed by Western blotting. We would suggest that this abnormally fucosylated form of haptoglobulin could be a useful tool in the clinical diagnosis of cancer patients.

### Comparison of 10 monoclonal antibodies to high molecular weight glycoprotein (MAM-6, EMA) by means of immunohistochemical large scale study

St. Zotter[1], A. Lossnitzer[1], K.D. Kunze[1], Ph.C. Hageman[2], J. Hilkens[2], J. Peterse[3] & J. Hilgers[2]

[1]*Inst. Path. Anat., Medical Academy, Dresden, GDR-8019;* [2]*Dept. Tumour Biology and* [3]*Dept. Pathology, The Netherlands Cancer Institute, Amsterdam, NL-1066 CX.*

Controversial data have been reported on the specificity of monoclonal antibodies (MoAbs) against the epithelial membrane antigen (EMA). In order to shed light on this problem, 10 MoAbs have been compared for their reactivity on serial sections of 165 formalin fixed and paraffin embedded tumours using the indirect immunoperoxidase technique. The MoAbs investigated were: antibodies to MAM-6 (epitopes a-f), HMFG-1 and HMFG-2 (provided by J. Taylor-Papadimitriou), NCRC-11 (M. Price), DF3 (D. Kufe) and E29 (anti-EMA, Dako). The tissue sample, consisted of 115 epithelial tumours (lung, breast, squamous tissue, colon, stomach, parotid gland, prostate gland, urinary bladder, kidney, ovary, uterus), 10 melanomas, 15 sarcomas, 10 brain tumours and 15 malignant lymphomas. All the antibodies proved to be useful reagents for the detection of epithelial tissue (between 80 and 95% of the specimens). However, the cross-reactivities observed with several sarcomas (preferentially those of myogenic nature) and with some lymphomas varied strikingly among the antibodies, the commercially available E29 being one of the MoAbs giving rise to most cross-reactions. Moreover, different staining patterns were observed on epithelial neoplasms stressing the different epitope (antigen?) specificity of the antibodies.

**Ribonucleotide reductase $M_1$ subunit as a marker of cellular proliferation**

G.J. Mann[1], E.A. Musgrove[1] & L. Thelander[2]

[1]*Ludwig Institute for Cancer Research, Sydney Branch, University of Sydney, NSW 2006, Australia and* [2]*Department of Biochemistry 1, Karolinska Institute, S-104 01 Stockholm, Sweden.*

Ribonucleotide reductase catalyses the first unique, rate-limiting step in DNA synthesis. We have measured the cell-cycle variation of its $M_1$ subunit using a monoclonal antibody (Engström *et al., EMBO J.*, **3**, 863, 1984) and indirect immunofluorescence flow cytometry in para-formaldehyde-fixed cells, with simultaneous measurement of DNA content. In logarithmically growing cultured cells (e.g. CCRF-CEM lymphoblasts) $M_1$ is present throughout the cell cycle in proportion to cell size. In contrast, there is no detectable $M_1$ in freshly isolated peripheral blood mononuclear cells (PBMC), but it appears within 24 h of mitogen stimulation. $M_1$ declines to very low levels in cultures grown to limiting density (B16 melanoma), the reduction being largely confined to cells with 2n ($G_0/G_1$) DNA content. HL-60 promyelocytic leukaemic cells, induced to terminally differentiate by dimethyl sulphoxide or 12-*o*-tetradecanoylphorbol-13-acetate, showed a similar marked decrease in $M_1$ content concomitant with the cessation of cell division. When logarithmically growing CCRF-CEM cells and fresh PBMC were mixed in varying proportions it was possible to discriminate between the two cell types according to the presence or absence of $M_1$. We conclude that this protein is retained during $G_1$ and only disappears when continuous cycling ceases (i.e. in $G_0$ cells). Its presence may thus more completely indicate the proportion of cycling cells (i.e. the growth fraction) in a mixed population than conventional, DNA labelling, techniques. Since $M_1$ may be detected without the prior administration of any agent ($^3$H-thymidine or bromodeoxyuridine), its measurement should be valuable in *in vivo* studies of tumour cell kinetics.

**The detection of drug resistant tumour cells using flow cytometry**

A. deFazio, E.A. Musgrove, N. Heneine & M.H.N. Tattersall

*Ludwig Institute for Cancer Research, University of Sydney, Sydney, NSW 2006, Australia*

Evidence suggests that drug resistance in tumours frequently arises as a consequence of spontaneous somatic mutation (Goldie & Coldman, *Cancer Treatment Rep.*, **63**, 1727, 1979). The measurement of mutation frequency could therefore be useful in cancer management as an index of the tumour's potential to become resistant to therapy, Because current techniques for cloning human solid tumour cells manifest low cloning efficiencies, they are unsuitable for the detection of rare spontaneous mutants. An alternative approach to this problem is the use of the thymidine analogue 5′-bromodeoxyuridine (BrUdR). Cells that continue to proliferate, i.e. incorporate BrUdR, in the presence of a selective agent are immunofluorescently stained using our anti-BrUdR monoclonal antibody. This small proportion of resistant cells is quantitated flow cytometrically. 6-Thioguanine (6TG) resistance, usually attributed to a lack of the enzyme hypoxanthine phosphoribosyltransferase (HPRT), was measured in two cell lines, CCRF-CEM (human T-cell leukaemia) and L1210 (mouse T-cell line). The frequency of BrUdR positive cells, after treatment with 6TG, was found to be $3.0\times10^{-5}$ and $3.3\times10^{-5}$ respectively. The biological significance of the BrUdR positive cells was validated by pre-treatment of cell samples with HAT medium, thus removing the HPRT population, and by comparison with results from cloning assays. The method has been successfully used with tumour cell lines and its application to human tumours is being assessed.

The organisers gratefully acknowledge the generous support of the Cancer Research Campaign and the Imperial Cancer Research Fund. In addition, the meeting has been kindly supported by the following commercial companies: Boehringer Ingelheim, Beecham Research, Bristol-Myers, Eli Lilly & Co., Farmitalia Carlo Erba, Glaxo Laboratories, Kirby-Warrick Pharmaceuticals, Lederle Laboratories, Roche Products, Wellcome Laboratories


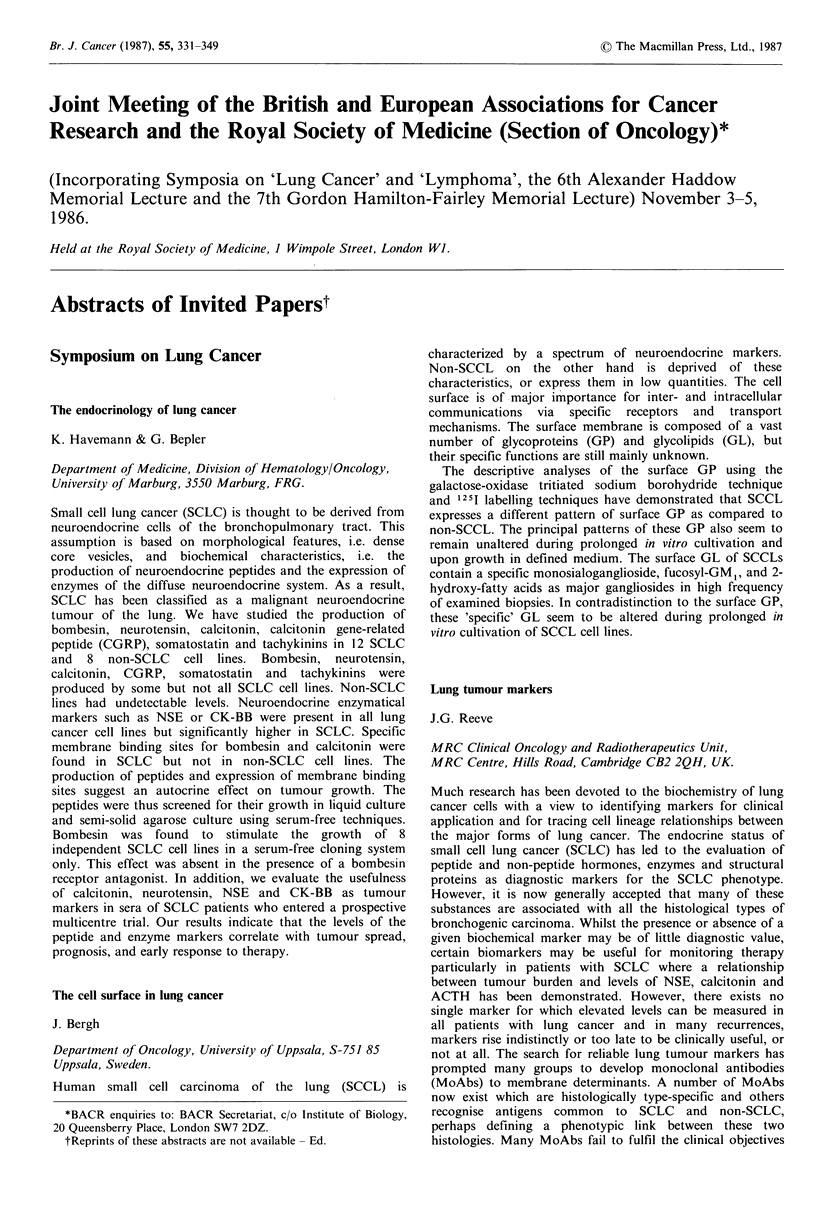

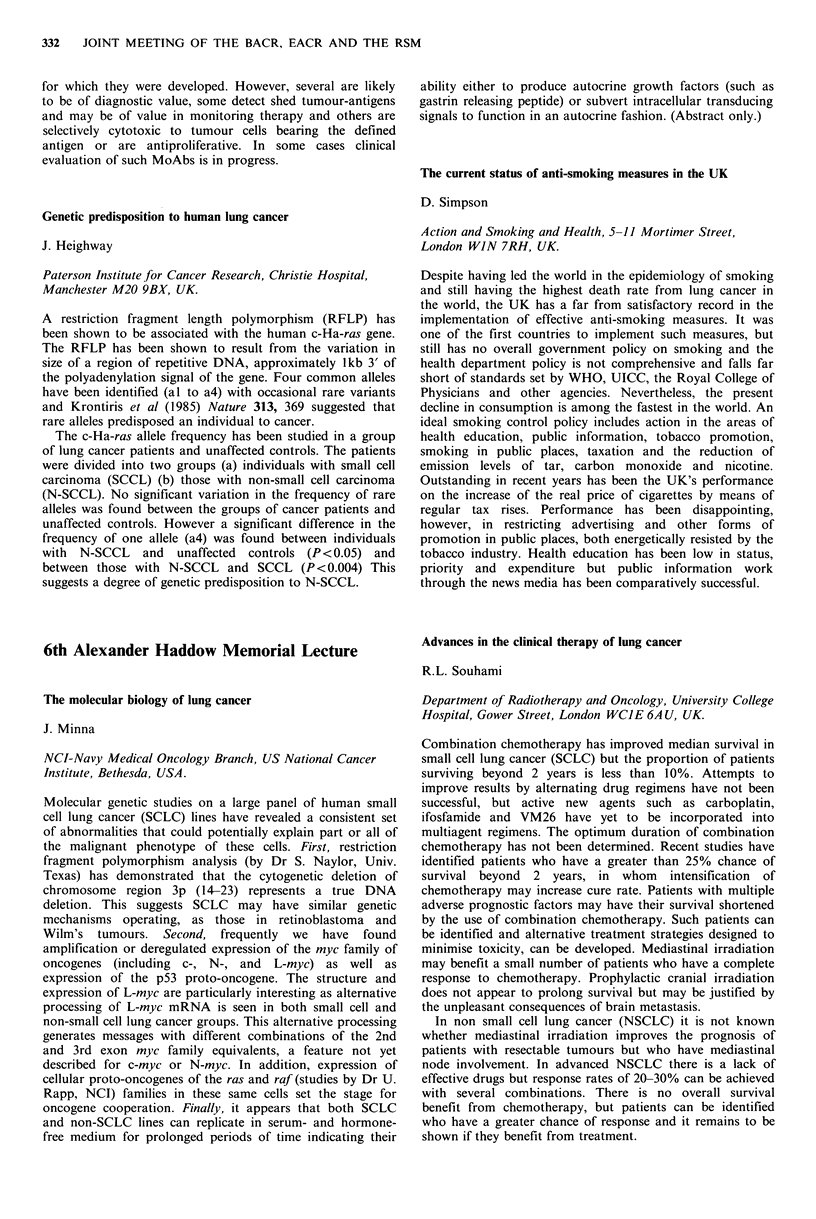

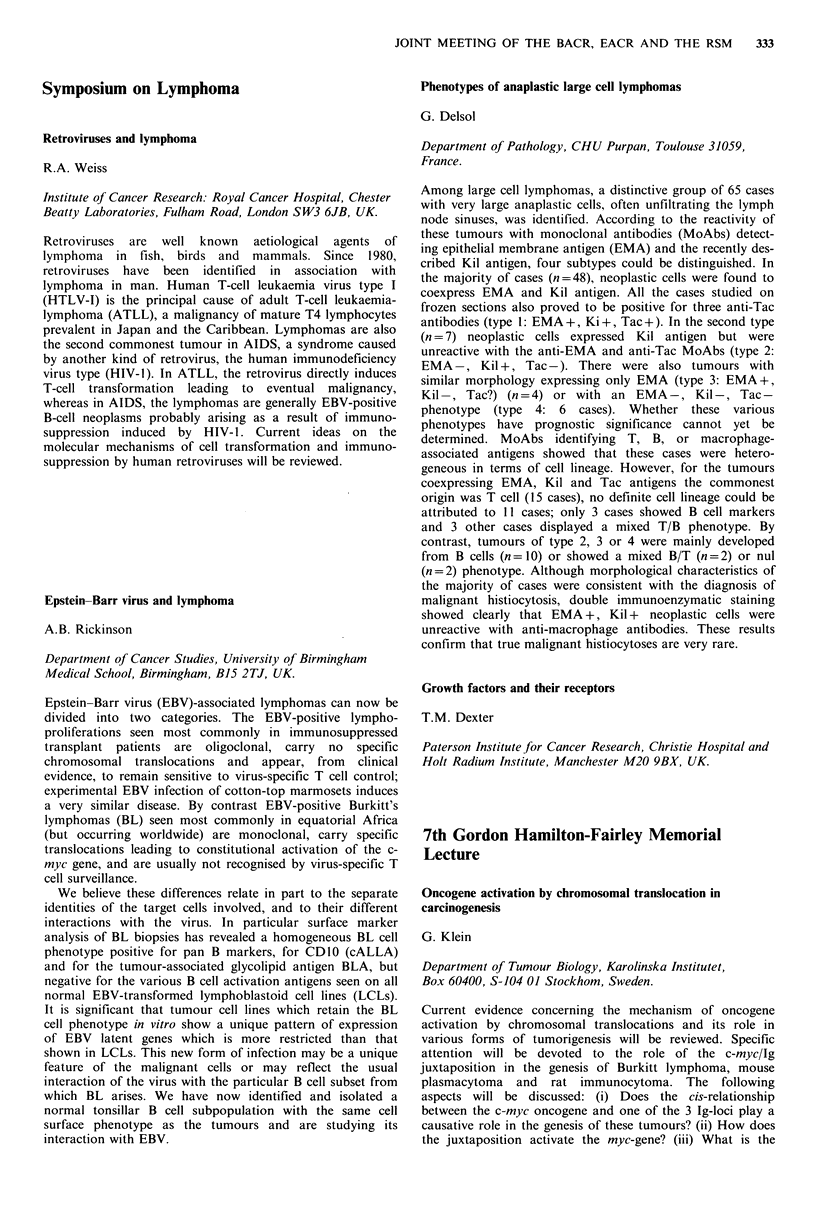

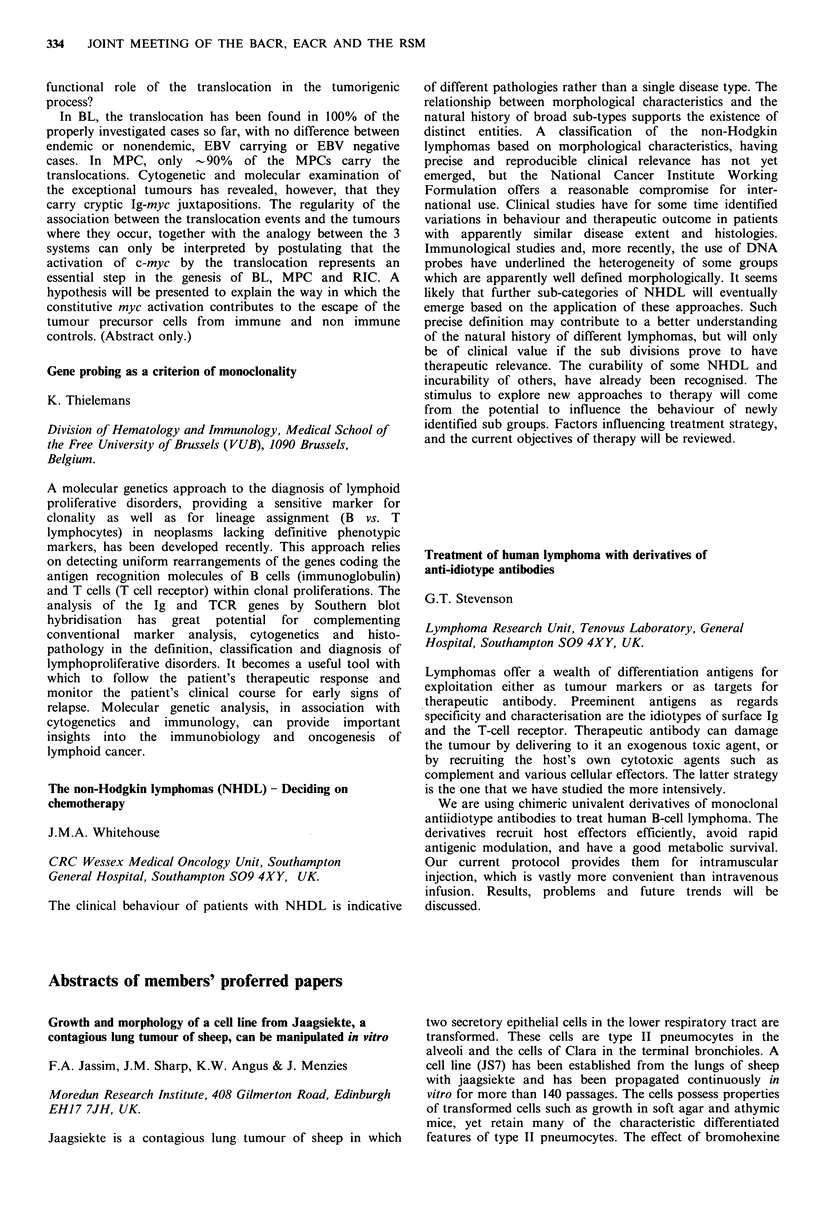

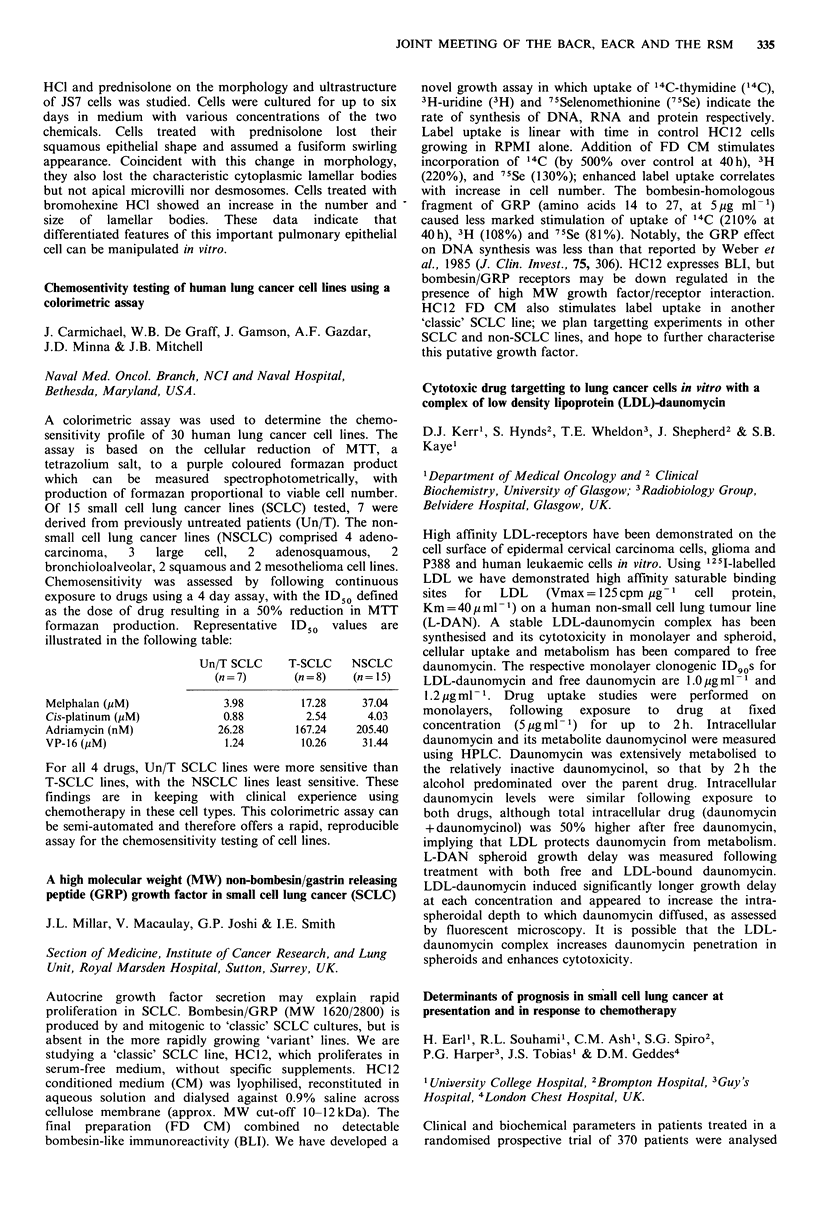

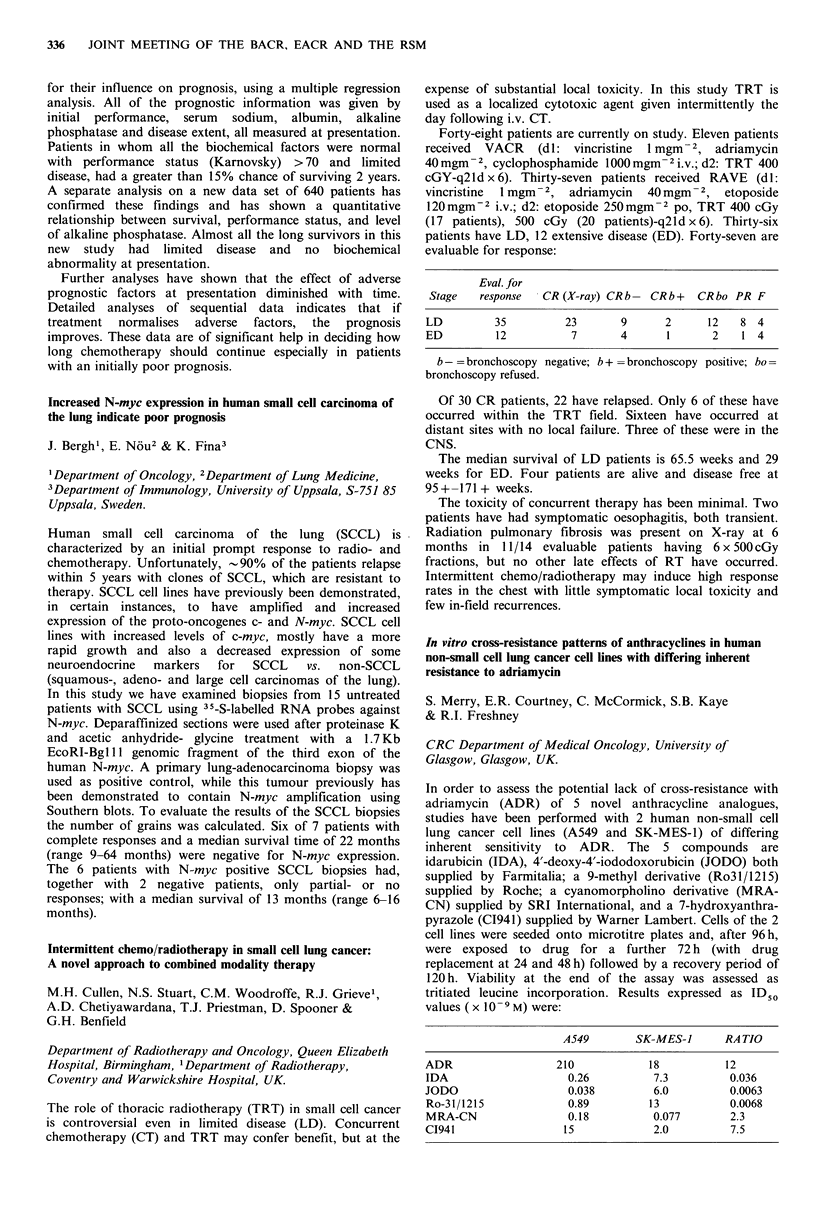

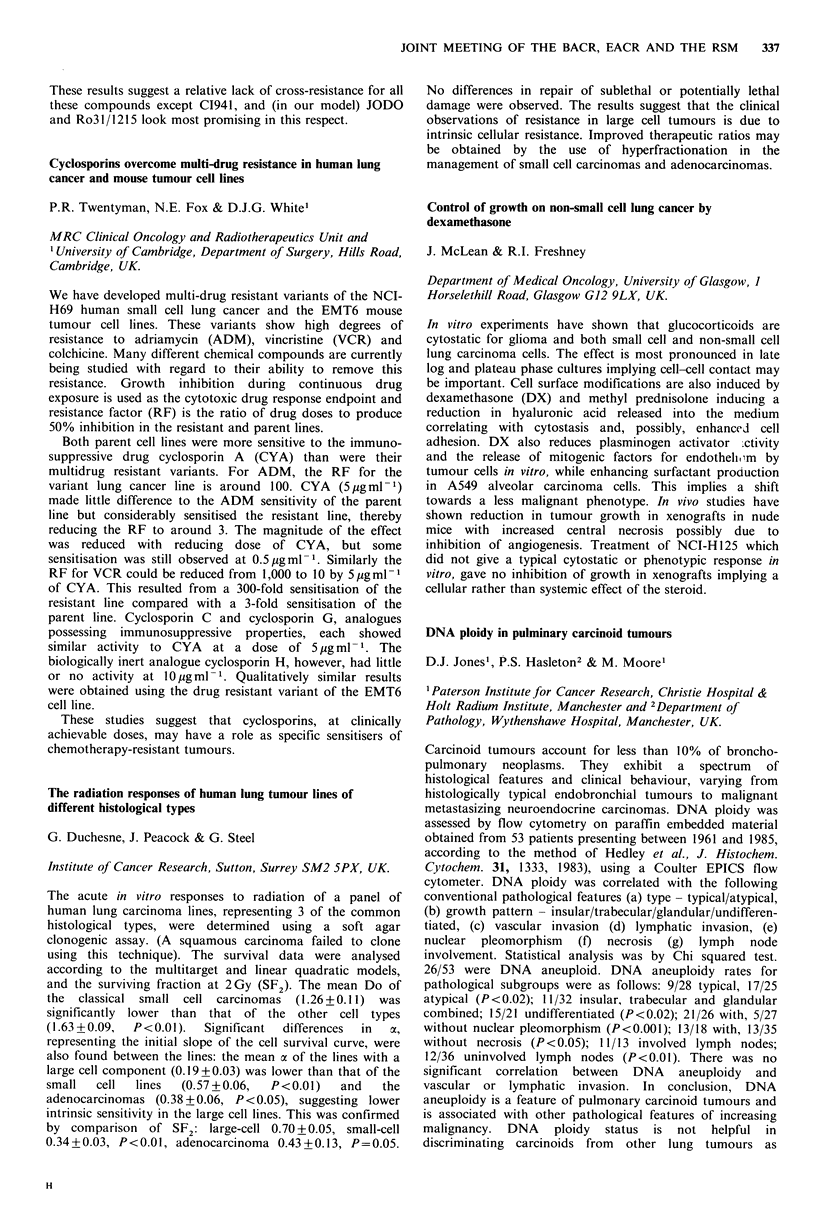

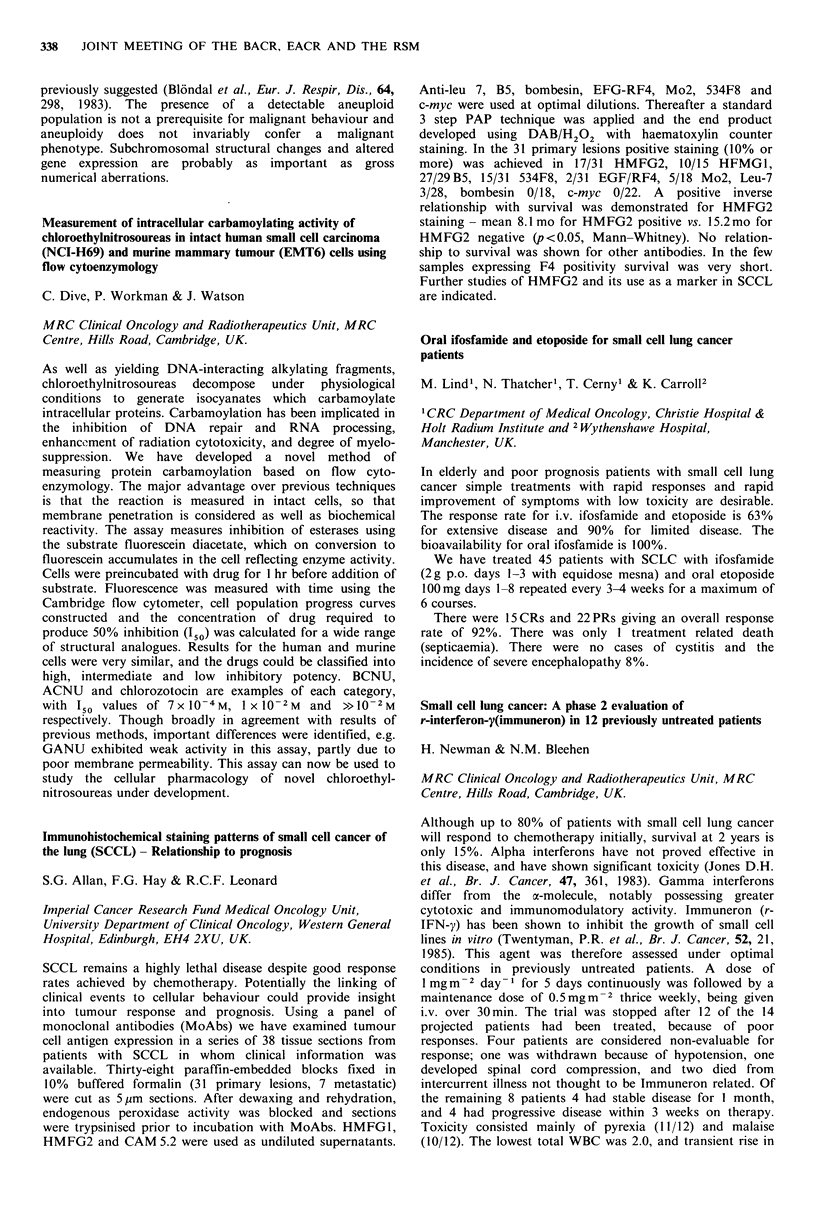

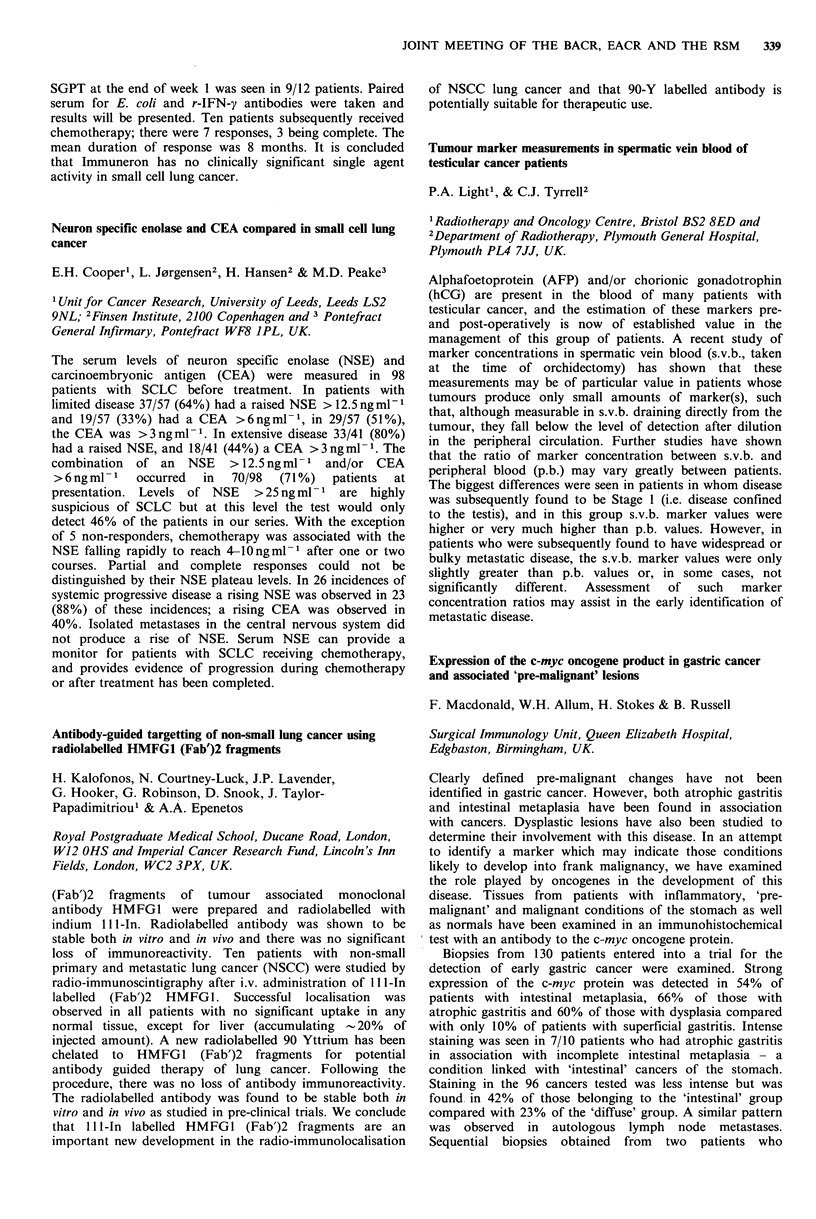

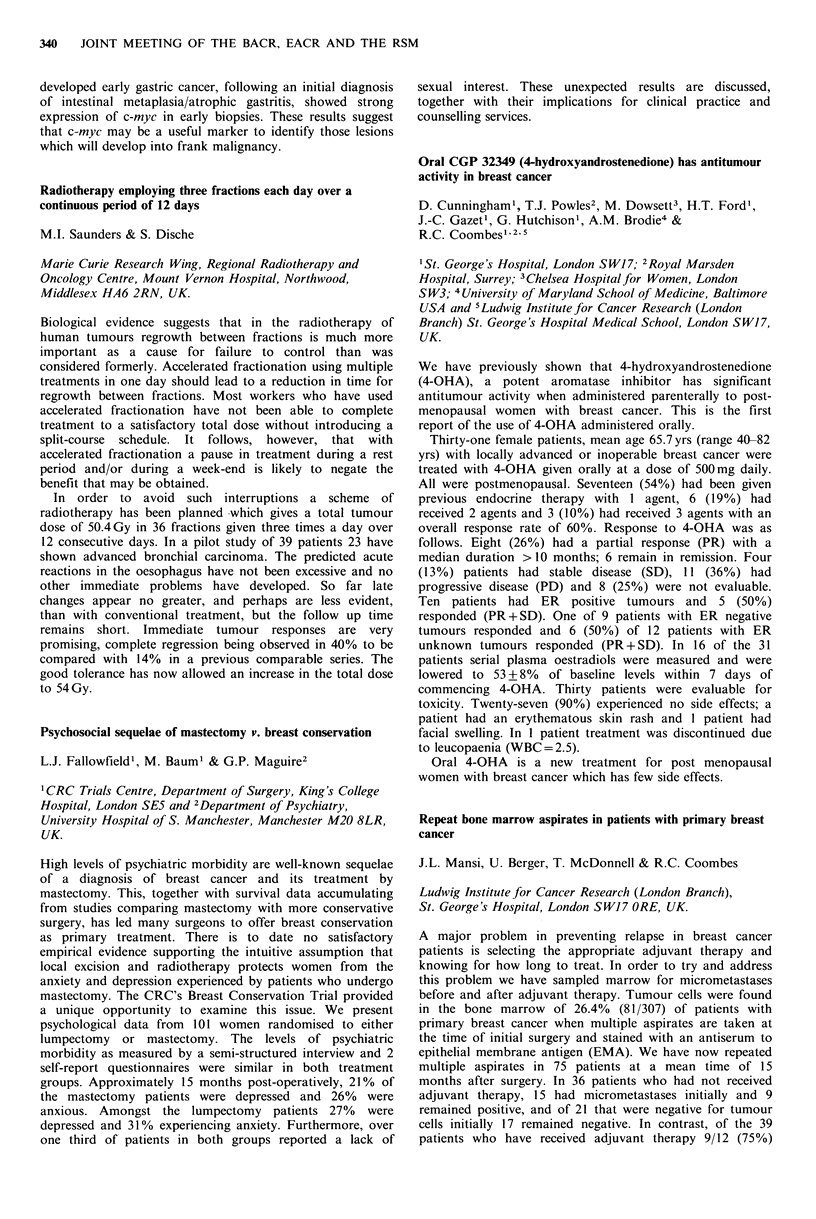

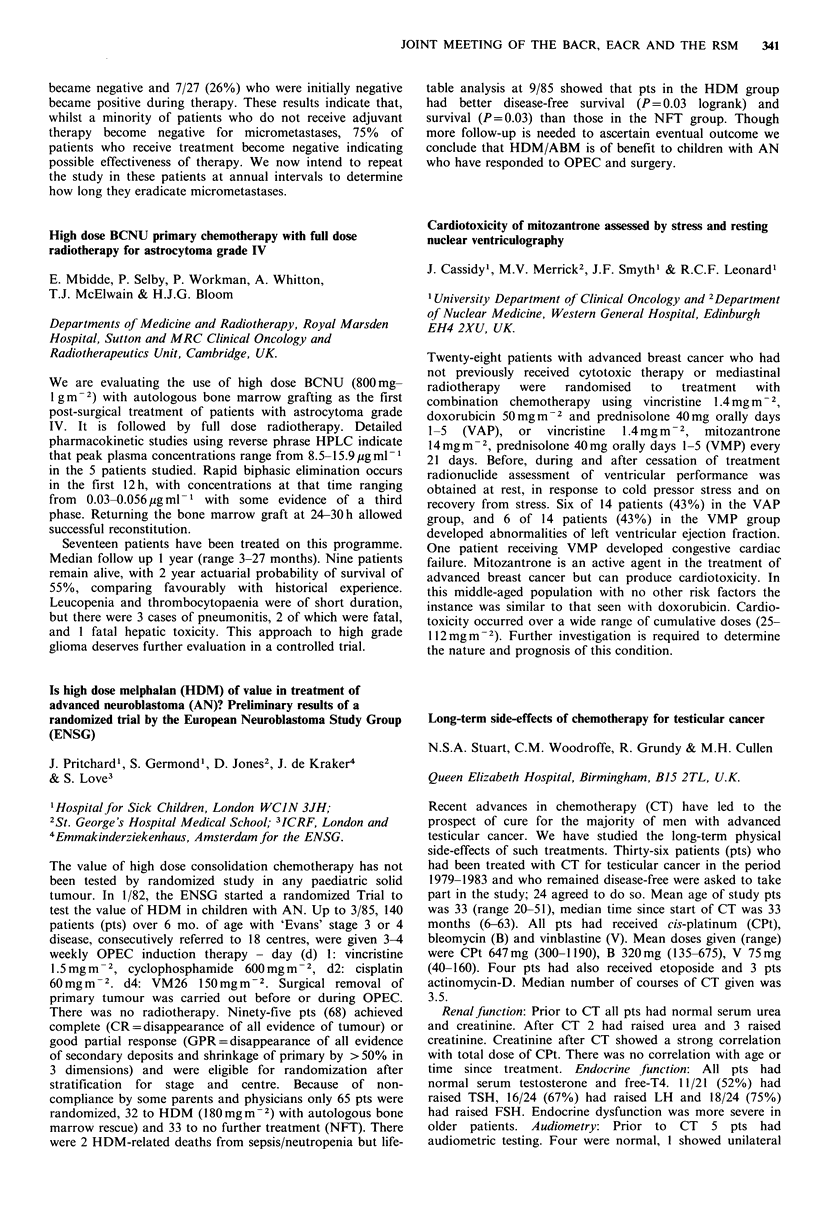

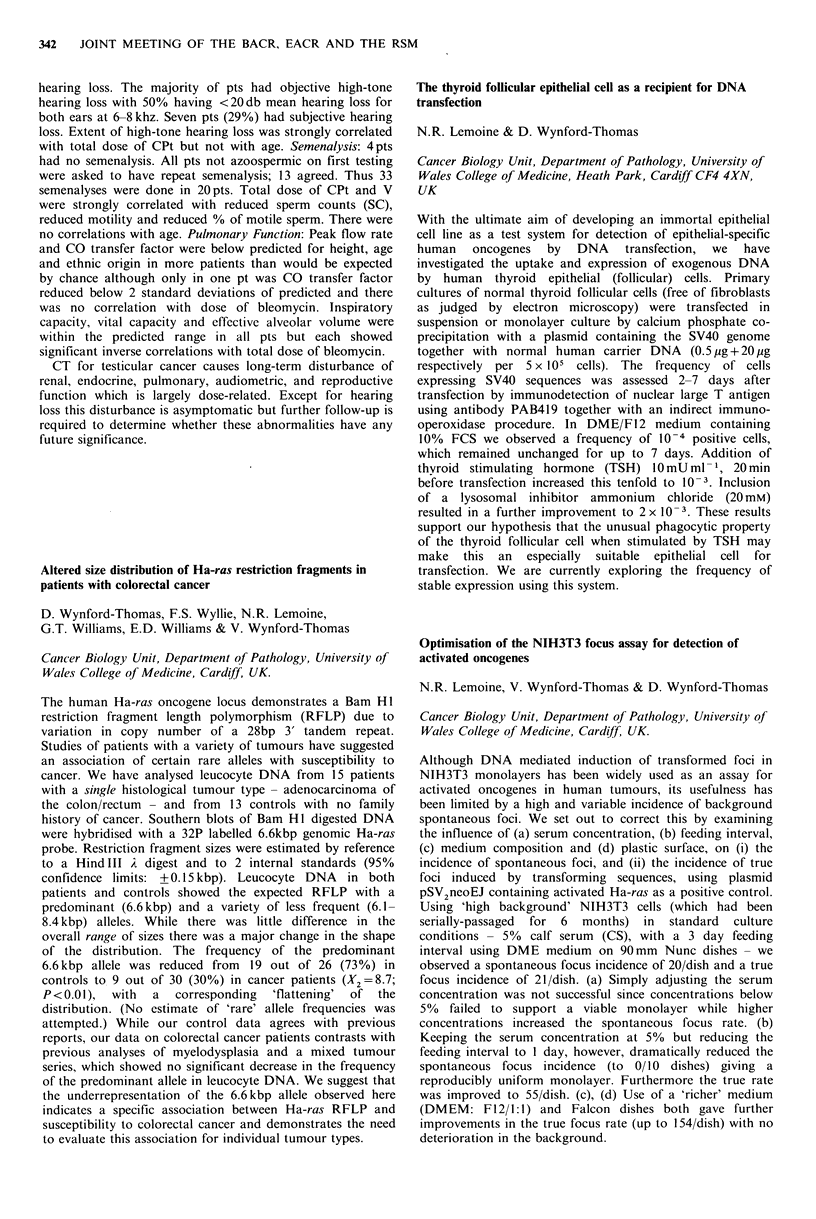

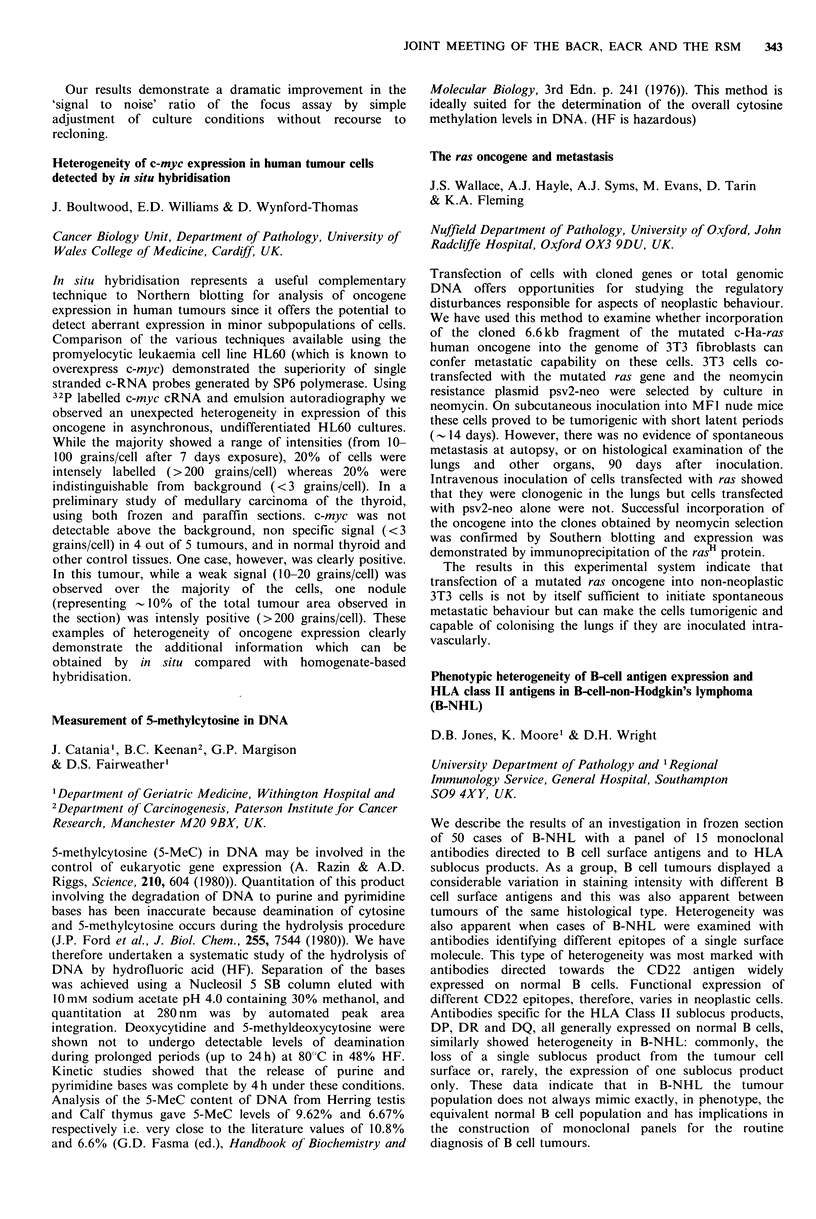

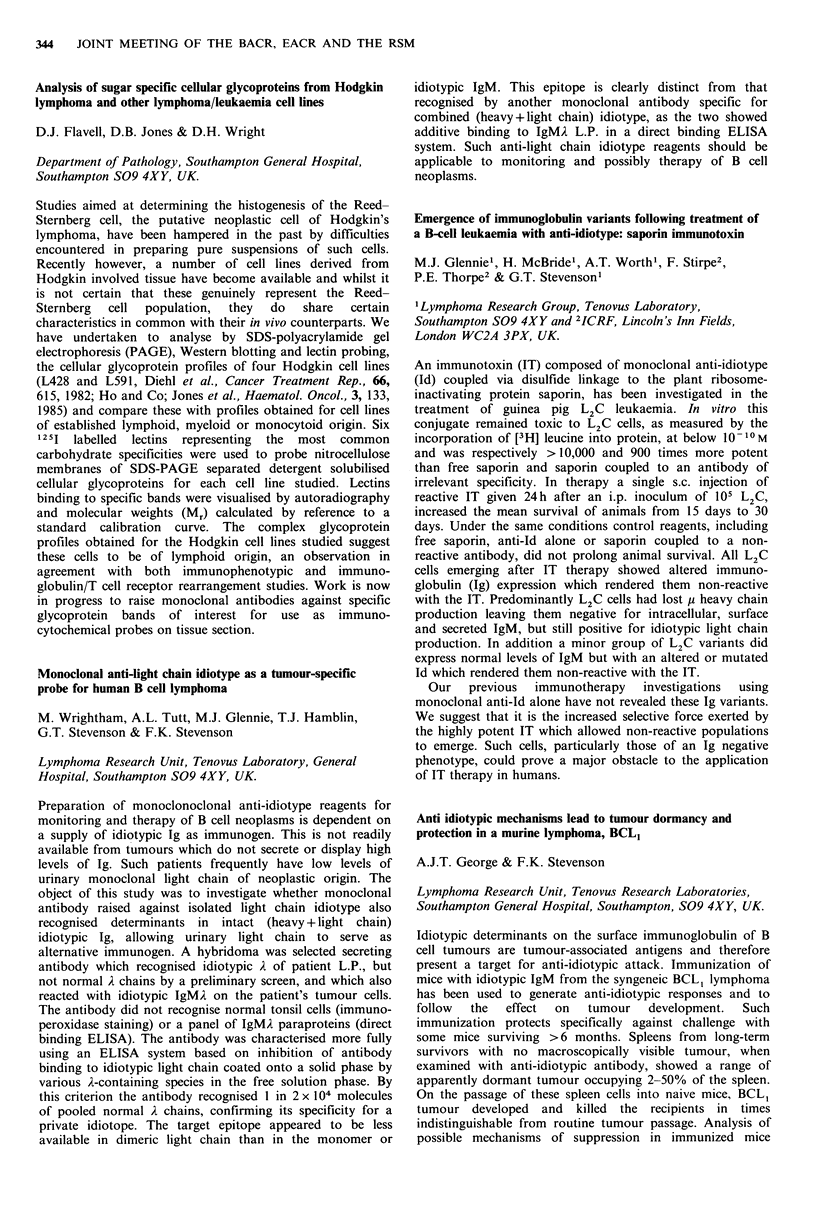

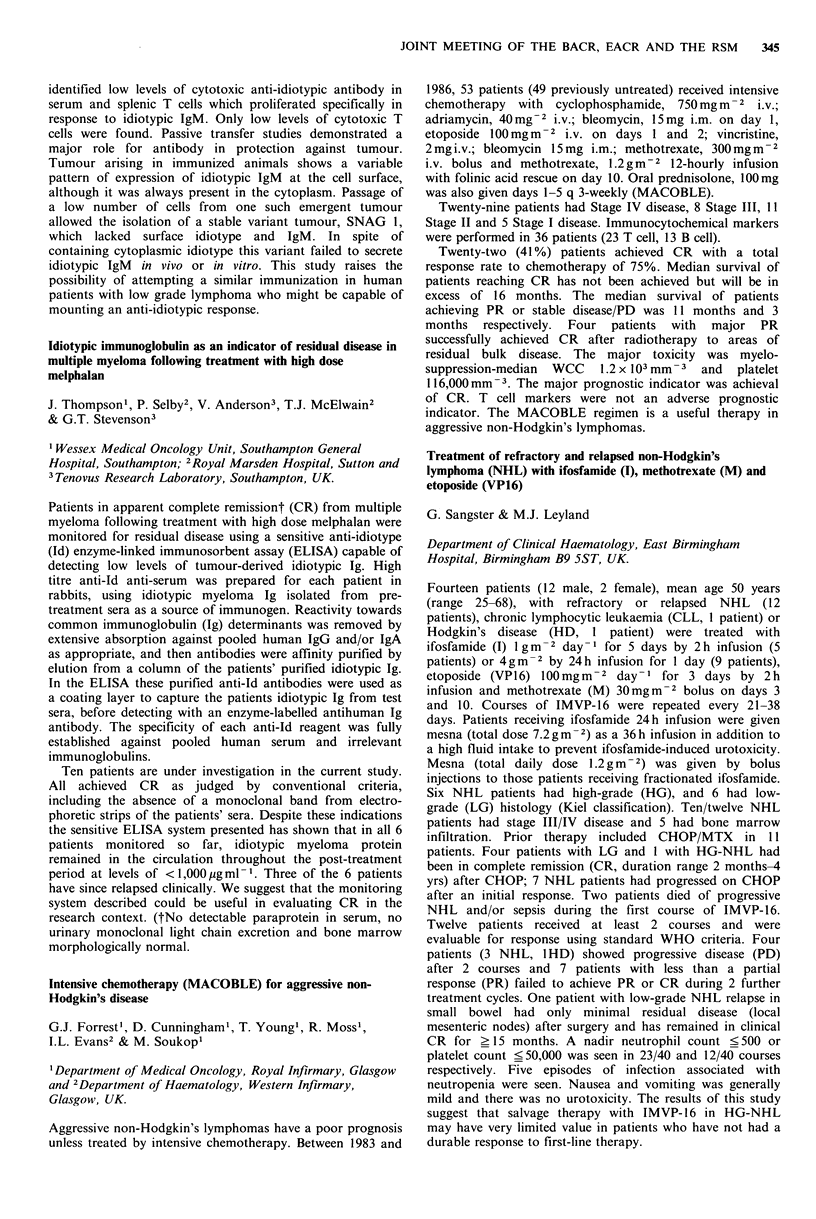

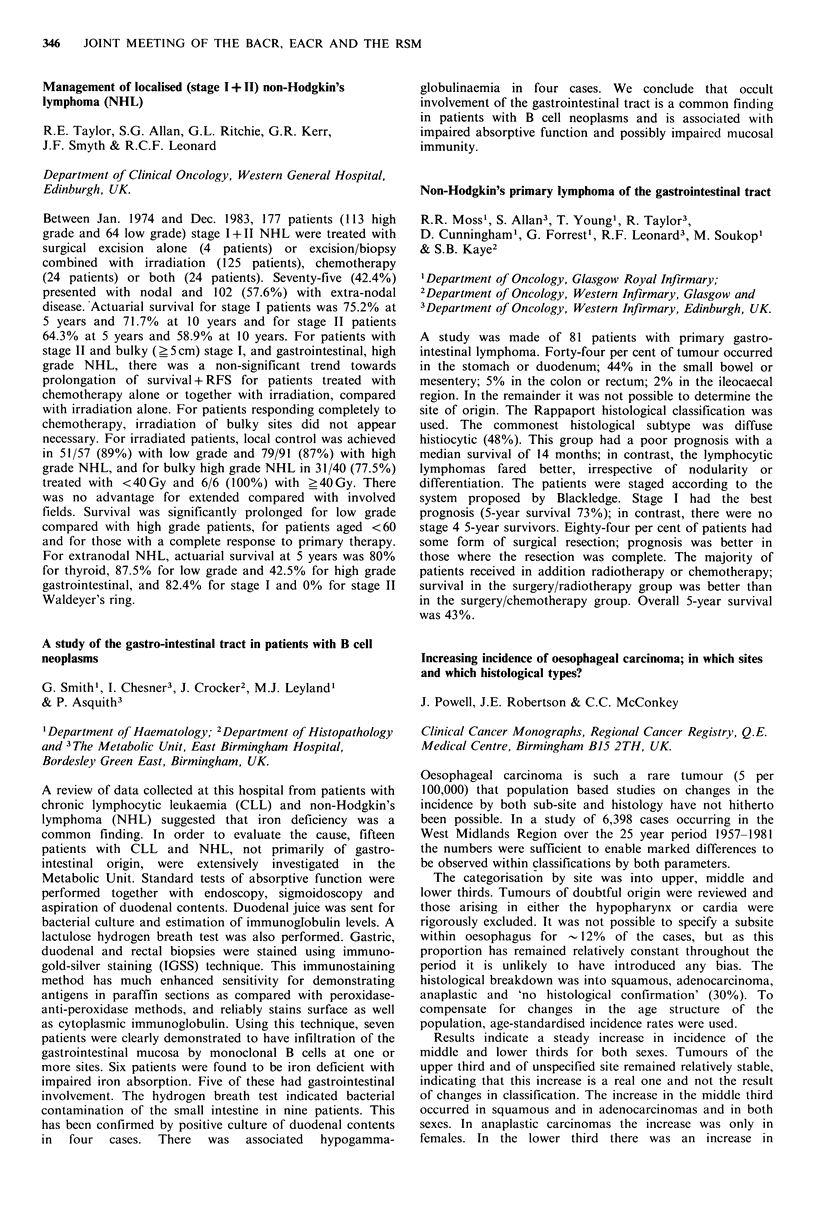

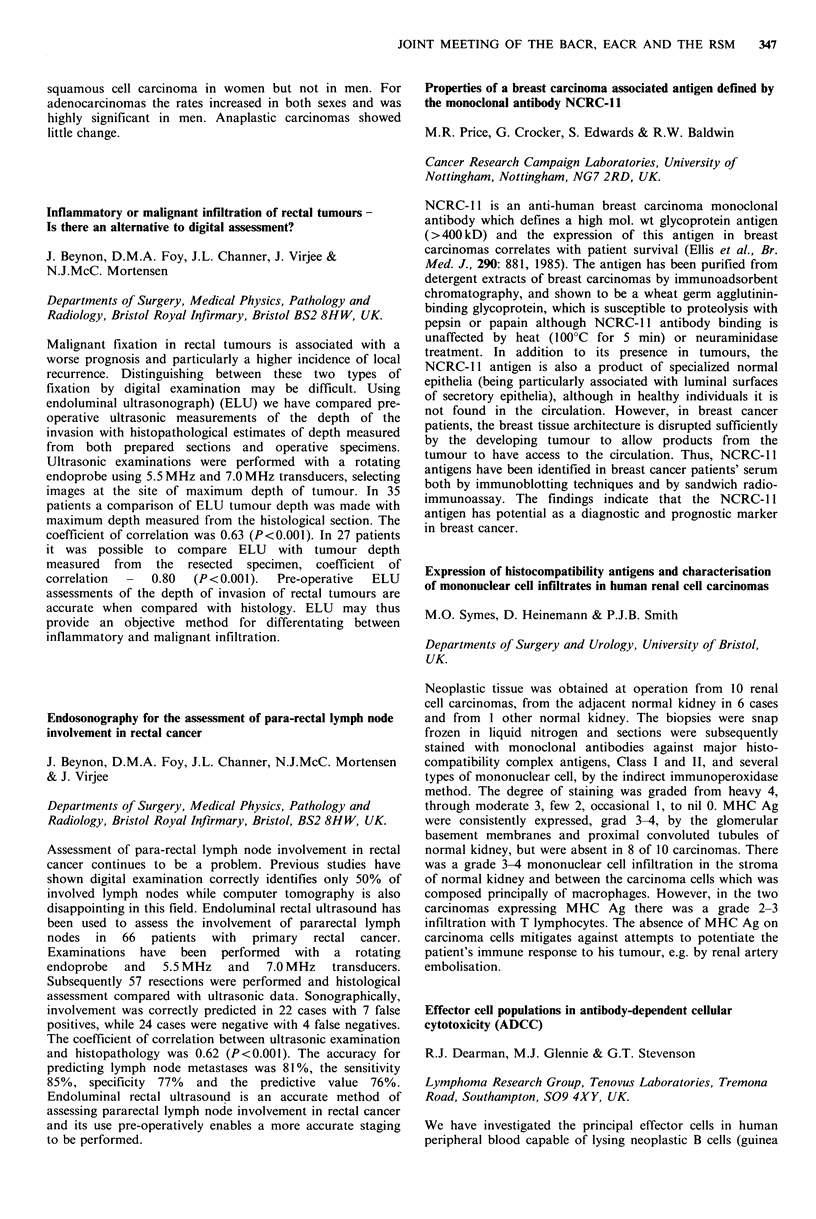

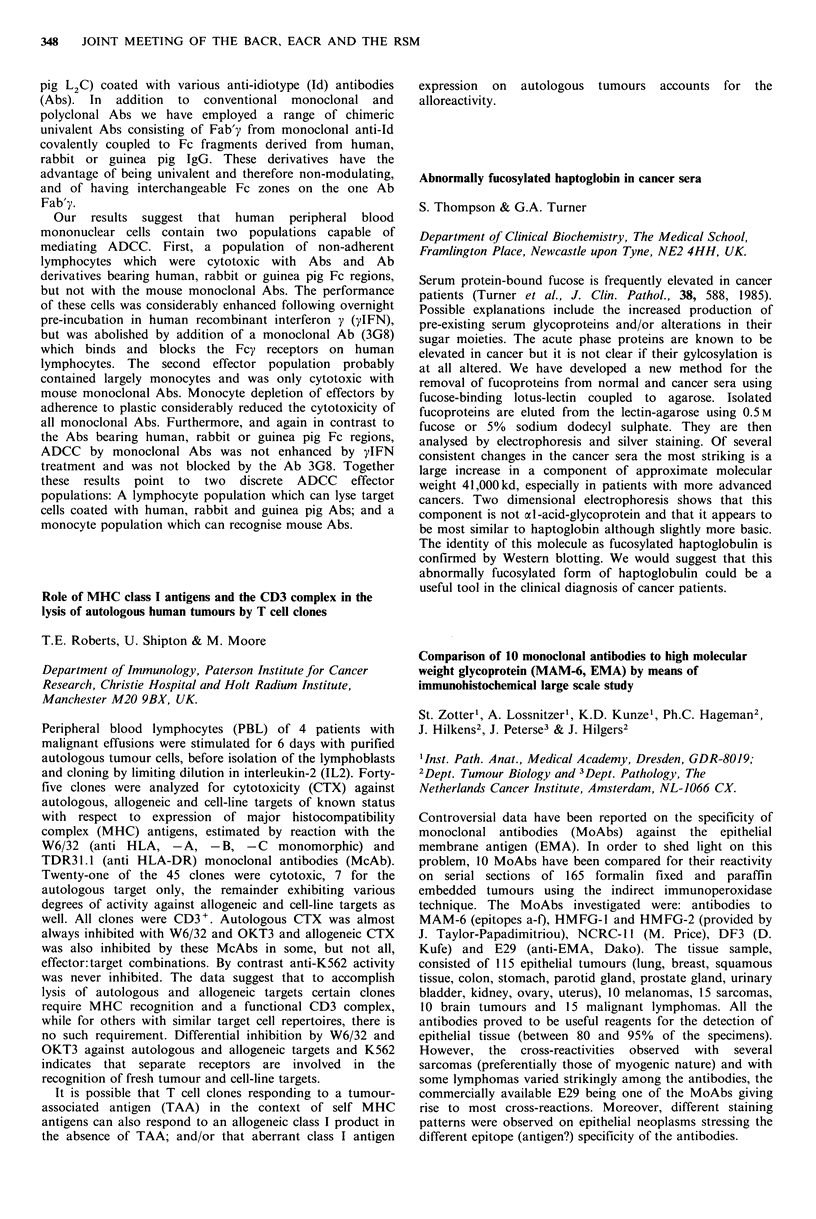

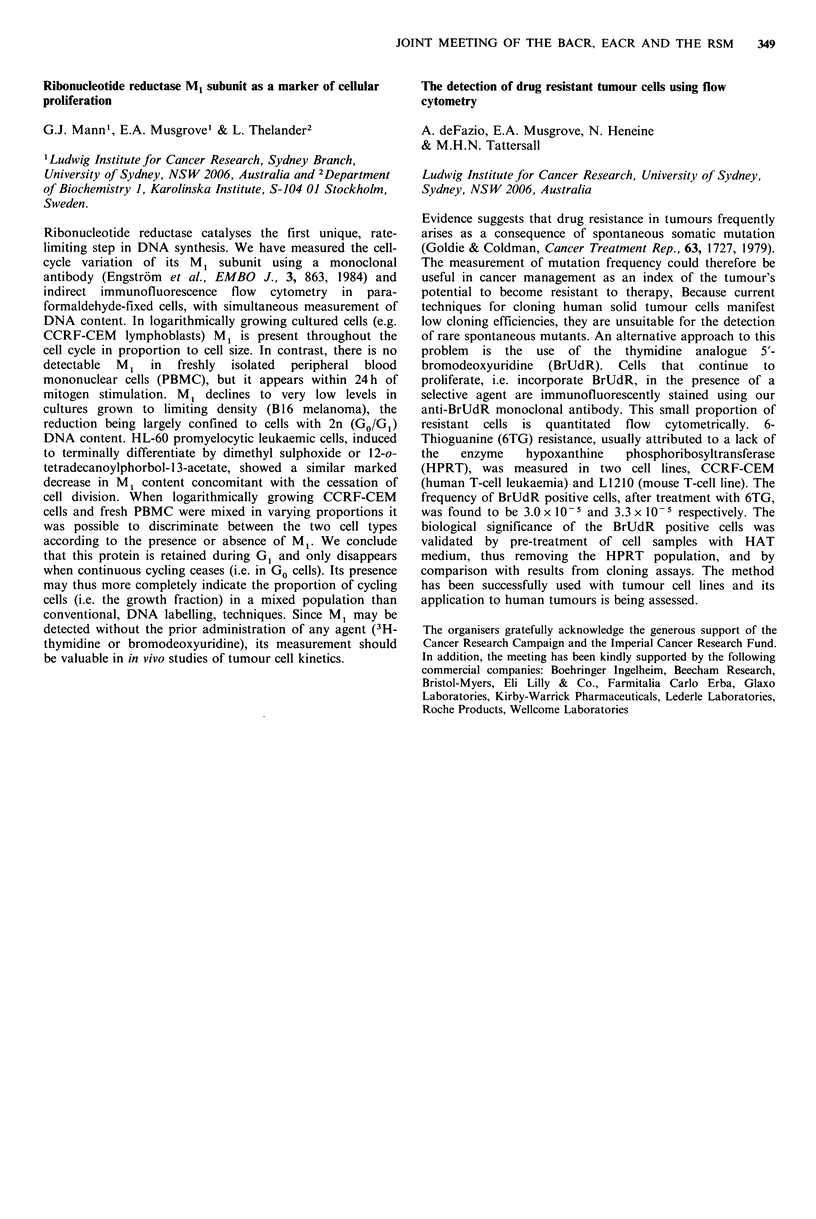


## References

[OCR_00473] Arklie J., Taylor-Papadimitrious J., Bodmer W., Egan M., Millis R. (1981). Differentiation antigens expressed by epithelial cells in the lactating breast are also detectable in breast cancers.. Int J Cancer.

[OCR_01010] Blöndal T., Arnorsson T., Bengtsson A., Wilander E. (1983). Nuclear DNA in carcinoid tumours of the lung.. Eur J Respir Dis.

[OCR_00481] Cordell J., Richardson T. C., Pulford K. A., Ghosh A. K., Gatter K. C., Heyderman E., Mason D. Y. (1985). Production of monoclonal antibodies against human epithelial membrane antigen for use in diagnostic immunocytochemistry.. Br J Cancer.

[OCR_00485] Delsol G., Gatter K. C., Stein H., Erber W. N., Pulford K. A., Zinne K., Mason D. Y. (1984). Human lymphoid cells express epithelial membrane antigen. Implications for diagnosis of human neoplasms.. Lancet.

[OCR_01924] Diehl V., Kirchner H. H., Burrichter H., Stein H., Fonatsch C., Gerdes J., Schaadt M., Heit W., Uchanska-Ziegler B., Ziegler A. (1982). Characteristics of Hodgkin's disease-derived cell lines.. Cancer Treat Rep.

[OCR_02444] Ellis I. O., Hinton C. P., MacNay J., Elston C. W., Robins A., Owainati A. A., Blamey R. W., Baldwin R. W., Ferry B. (1985). Immunocytochemical staining of breast carcinoma with the monoclonal antibody NCRC 11: a new prognostic indicator.. Br Med J (Clin Res Ed).

[OCR_02695] Engström Y., Rozell B., Hansson H. A., Stemme S., Thelander L. (1984). Localization of ribonucleotide reductase in mammalian cells.. EMBO J.

[OCR_00496] Epenetos A. A., Canti G., Taylor-Papadimitriou J., Curling M., Bodmer W. F. (1982). Use of two epithelium-specific monoclonal antibodies for diagnosis of malignancy in serous effusions.. Lancet.

[OCR_00508] Fisher E. R., Palekar A., Rockette H., Redmond C., Fisher B. (1978). Pathologic findings from the National Surgical Adjuvant Breast Project (Protocol No. 4). V. Significance of axillary nodal micro- and macrometastases.. Cancer.

[OCR_00502] Fisher E. R., Swamidoss S., Lee C. H., Rockette H., Redmond C., Fisher B. (1978). Detection and significance of occult axillary node metastases in patients with invasive breast cancer.. Cancer.

[OCR_01817] Ford J. P., Coca-Prados M., Hsu M. T. (1980). Enzymatic analysis of 5-methylcytosine content in eukaryotic DNA. Study of intracellular Simian Virus 40 DNA.. J Biol Chem.

[OCR_02703] Goldie J. H., Coldman A. J. (1979). A mathematic model for relating the drug sensitivity of tumors to their spontaneous mutation rate.. Cancer Treat Rep.

[OCR_00982] Hedley D. W., Friedlander M. L., Taylor I. W., Rugg C. A., Musgrove E. A. (1983). Method for analysis of cellular DNA content of paraffin-embedded pathological material using flow cytometry.. J Histochem Cytochem.

[OCR_00277] Krontiris T. G., DiMartino N. A., Colb M., Parkinson D. R. Unique allelic restriction fragments of the human Ha-ras locus in leukocyte and tumour DNAs of cancer patients.. Nature.

[OCR_00514] PICKREN J. W. (1961). Significance of occult metastases. A study of breast cancer.. Cancer.

[OCR_01806] Razin A., Riggs A. D. (1980). DNA methylation and gene function.. Science.

[OCR_00518] Redding W. H., Coombes R. C., Monaghan P., Clink H. M., Imrie S. F., Dearnaley D. P., Ormerod M. G., Sloane J. P., Gazet J. C., Powles T. J. (1983). Detection of micrometastases in patients with primary breast cancer.. Lancet.

[OCR_00523] Rosen P. P., Saigo P. E., Braun D. W., Weathers E., DePalo A. (1981). Predictors of recurrence in stage I (T1N0M0) breast carcinoma.. Ann Surg.

[OCR_00528] Rosen P. P., Saigo P. E., Braun D. W., Weathers E., Fracchia A. A., Kinne D. W. (1981). Axillary micro- and macrometastases in breast cancer: prognostic significance of tumor size.. Ann Surg.

[OCR_00533] Sloane J. P., Ormerod M. G., Imrie S. F., Coombes R. C. (1980). The use of antisera to epithelial membrane antigen in detecting micrometastases in histological sections.. Br J Cancer.

[OCR_00539] Taylor-Papadimitriou J., Peterson J. A., Arklie J., Burchell J., Ceriani R. L., Bodmer W. F. (1981). Monoclonal antibodies to epithelium-specific components of the human milk fat globule membrane: production and reaction with cells in culture.. Int J Cancer.

[OCR_00546] Viac J., Reano A., Brochier J., Staquet M. J., Thivolet J. (1983). Reactivity pattern of a monoclonal antikeratin antibody (KL1).. J Invest Dermatol.

[OCR_00551] Wells C. A., Heryet A., Brochier J., Gatter K. C., Mason D. Y. (1984). The immunocytochemical detection of axillary micrometastases in breast cancer.. Br J Cancer.

[OCR_00490] de Mascarel I., Trojani M., Abadjian G., Durand M., Bonichon F., Coindre J. M., Meuge-Moraw C. (1982). Ganglions axillaires dans les cancers du sein. Comparaison des techniques d'analyse histologique standard et en coupes macroscopiquement sériées.. Bull Cancer.

